# Supplementary material for: Human pathogenic RNA viruses establish noncompeting lineages by occupying independent niches
Source: Proc Natl Acad Sci U S A. 2022 May 31;119(23):e2121335119. doi: 10.1073/pnas.2121335119 (PMC9191635; doi:10.1073/pnas.2121335119)
Supplement: Supplementary File [file pnas.2121335119.sapp.pdf]

PNAS

Supplementary Information for

Human pathogenic RNA viruses establish non-competing lineages by occupying independent niches

Pascal Mutz, Nash D. Rochman, Yuri I. Wolf, Guilhem Faure, Feng Zhang, and Eugene V. Koonin

Nash D. Rochman
Email: nash.rochman@nih.gov

Feng Zhang
Email: zhang@broadinstitute.org

Eugene V. Koonin
Email: koonin@ncbi.nlm.nih.gov

This PDF file includes:

Extended Methods
Declarations
SI References
List of abbreviations
Figures S1 to S41

Other supplementary materials for this manuscript include the following:

The datasets can be found

https://doi.org/10.5281/zenodo.5711959
or
https://ftp.ncbi.nih.gov/pub/wolf/_suppl/virNiches/

This repository includes:

Tables

Table S1: Virus characteristics including abbreviation, family, order, tax id, sequence length threshold (for nearly complete genomes), vaccination status, mode of transmission, circulation (human or human/zoonotic), available treatment, and disease progression (acute or chronic).

Table S2: Sequence IDs of outgroup constituents.

Table S3: GISAID acknowledgements for the SARS-CoV-2 sequences used in this study.

Table S4: Summary of manual sequence curation indicating lab- or vaccine-related keywords used to prune sequences.

Table S5: Sequence IDs for EVA and H3N2 e10, e100, d10, and d100 subtrees.

Table S6: Sequence IDs for ML and GL lineages.

Table S7: Sequence IDs for the H3N2 “new” and “old” subtrees.

Table S8: Mutation rates (in units of nucleotide substitutions per site per year) and estimated dates for the last common ancestor of all tree/subtrees. Negative dates represent years prior to 0 CE.

Table S9: Mean dN/dS values.

Table S10: Yearly viral cases and generation time estimations (with references).

Table S11: Metadata including the sequence ID, host, date of isolation, location of isolation, and subtype.

Table S12: Overview of key parameters including number of GLs N, Ne, and MRCA for each virus species as well as the mean over all species.

Directories

Alignments: ORFeome alignments (excluding stop codons) with the exception of SARS-CoV-2.

Correlated Subtrees: Subtrees representing all correlated-clades (genealogical lineages; GL) in Newick format.

Diversity TMRCA and Skyline Plots: .png files.

e/d/n/o Subtrees: EVA and H3N2 subtrees, evenly and diversely sampled as well as “early” and “late” subtrees for H3N2.

Genealogical Trees: Including global topologies (.main), subtrees, and grafted trees (.grafted).

Maximally Diverse Subtrees: Genealogical trees sampled to contain the same number of leaves as each respective tree in the Simulated Trees directory.

ORF References: The first and last nucleotide of each ORF in the reference sequence.

Redundancy Tables: List of all redundant isolates and the corresponding representative in the ORFeome-unique alignment.

Reference Sequences: Genbank page for all reference sequences.
Rooted Trees Global topologies for each virus.

Simulated Trees: Neutral models, based on Yule-Harding, for each virus.
Ultrametric Trees For each global topology.

Extended Methods

Multiple Sequence Alignments of RNA Virus Genomes

Nearly complete viral genomes were retrieved for all viruses except influenza A virus H3N2 and SARS-CoV-2 from NCBI virus (1) with the following minimal length requirements on nucleotide sequence (27000: MERS, BCoV1; 1700: Ebola; 15500: MMV; 12000: HRSV, HRV3, MRV; 11000: HMPV; 9500: TBEV; 8500: DENV, CHIKV, HCV, WNV, YFV, ZIKV; 5500: EVA, EVD, Norwalk, PeVA, SV; 5600: EVB, RVA; 6000: EVC; 4700: OHVA; 1200: HDV;). Genomes were retrieved on 10/28/2020, except for MERS, which was retrieved on 12/01/2020. Isolates from non-human hosts were included to take into account vector transmission and putative zoonotic reservoirs. Members of related viral families were used to construct an outgroup if sequence identity of the largest protein between outgroup candidate and main group was at least 30%. Up to 20 diverse reference viruses were selected among the outgroup candidates. Outgroup sequence IDs are listed in Supplementary Data, Table S2. For HDV and HRSV, no outgroup was available meeting these criteria.

Samples for Influenza A virus H3N2 (flu H3N2) segment HA were retrieved from the NCBI flu database (2) on 05/18/2021. Only (nearly) complete HA genome segments were considered (larger than 1600 nucleotides). No outgroup was considered forH3N2.

Due to the exceptionally large number of SARS-CoV-2 genomes sequenced by 2021, the SARS-CoV-2 tree and alignment analyzed in this work was subsampled from a larger alignment consisting of all high quality genomes that were available as of January 8, 2021 in the GISAID database(3), as previously described (4). Subsampling was conducted to maximize the sequence diversity (based on Hamming distance). Acknowledgments for the GISAID deposited sequences used in this study are displayed in Supplementary Data, Table S3.

Subalignments were considered for H3N2 and EVA, principally for the purpose of effective population size analysis (see below). For each, subalignments were constructed to sample an even number of isolates from every year at 10 and 100 isolates per year with three randomly sampled replicates (6 alignments in total). Two additional alignments were constructed to maximize the sequence diversity (based on Hamming distance, as for SARS-CoV-2) over the same number of isolates as the first set of subalignments given 10 and 100 isolates per year respectively.

In all cases sequences were harmonized to DNA (e.g. U was transformed to T to amend software compatibility) and aligned with MAFFT(5), using default settings.

Sequences were clustered according to 100% identity with no coverage threshold using CD-HIT (6), and otherwise default settings for MERS and H3N2. The longest sequence from each cluster was selected as a representative. For all other viruses, all sequences were considered for the next steps. Exterior ambiguous characters (preceding/succeeding the first/last defined nucleotide) were removed, and sequences with more than 10 remaining interior, completely ambiguous characters (“N”) were discarded. MAFFT was run with default settings to align the cleared unique sequences. Outliers based on hamming distance to the nearest neighbor and consensus were identified and removed from the set. Remaining sequences were aligned with MAFFT, default settings, together with the respective outgroup.

Note that, in principle, the redundancy introduced by this clustering step can introduce ambiguity in mapping metadata on the tree. We show that this is not an issue for these datasets below.

Exterior ambiguous characters (preceding/succeeding the first/last defined nucleotide) were removed. Sites corresponding to protein-coding ORFs were then mapped to the alignments from the reference sequences excluding stop codons (see Supplementary Data). Noncoding regions were discarded.

These resulting alignments contained out-of-frame gaps. We first identified gaps in the reference sequences corresponding to multiples of three nucleotides present in no more than 90% of all sequences (and thus likely corresponding to “true” insertions relative to the reference at the same position in at least 10% of all sequences). Sites corresponding to less common insertions and non-triplet gaps in the reference were discarded. The remaining gaps represent deletions relative to the reference sequence. Similarly, codons in the frame of the reference sequences where fewer than 10% of sequences in any of the three sites were represented by non-gap characters were removed. Gaps shorter than three nucleotides in the remaining sites were replaced with the

ambiguous character N. Longer gaps were shifted into frame and padded with ambiguous characters on either end of the gap to minimize the number of characters changed. Sequences composed of more than 25% gaps were then discarded.
Outliers among the remaining sequences were then identified based on the Hamming distance (excluding gaps and ambiguous characters) to the nearest neighbor and removed
Unique alignment rows were then identified, and redundant rows were discarded. Such redundancy, in addition to the redundancy introduced by prior clustering for MERS and H3N2, can introduce ambiguity in mapping metadata on the tree as a single, random representative isolate is selected to correspond to each unique row. As discussed above, the SARS-CoV-2 data were subsampled and matched with representative metadata accordingly. Among the remaining twenty-six alignments, only 7 had more than 10% of all isolates removed due to redundancy: Ebola (37%), EVC (15%), HRSV (13%), MERS (16%), MMV (20%), MRV (25%), and H3N2 (42%). For EVC, all but 5 redundant sequences mapped to rows in the alignment which were later pruned (see the following section on pruning lab-related isolates and select isolates from non-human hosts). For the remaining 6 it was confirmed that the sequencing dates for the redundant isolates correspond to those of the representative isolates mapped to the tree (see Figure S30) and thus the selection of these representatives does not introduce significant ambiguity. Tables listing all redundant sequence IDs and the sequence ID representing each redundant sequence in the respective alignment are available in the Supplementary Data.

Pruning Lab-Related Isolates from Non-Human Hosts

Samples related to laboratory experiments, vaccine-related sequences and patents were pruned based on an automated keyword search (see Supplementary Data, Table S4) followed by manual curation. Further, samples related to vaccine-derived poliomyelitis were removed from the EVC set as well as samples related to patients under treatment with direct-acting antivirals from the HCV set. The supplementary material contains analysis for trees maintaining “every” sequence (EVCe, HCVe) as well as only the “reduced” (EVCr, HCVr) set. Main figures display results for the reduced set.

Selecting subalignments for EVA and H3N2

In order to analyze the effect of sampling efforts on the effective population size (see below), several subalignments were generated for EVA and H3N2. To maintain an even representation over time, up to either 10 or 100 isolates (not unique sequences) were randomly selected (“even”/ “e”). To construct subalignments maximizing sequence diversity, we collected the same number of sequences as each evenly sampled subalignment through an iterative process maximizing the minimum hamming distance at each step (“diverse”/ ”d”). The complete alignment was reduced to the samples within each subset (keeping the outgroup) to retrieve eight final subalignments per virus (3x e_10, 3x e_100 and 1x d_10 and d100). The corresponding sequence IDs for each subalignment can be found in Supplementary Data, Table S5.

Metadata Analysis

Dates and locations of isolation are available for many isolates reported as calendar dates and city or country/administrative region of origin. The fraction of isolates for which metadata is available varies across the alignments. Calendar dates may be reported as the day, month, or only year. If the day or month was not specified, the collection date was assumed to be the midpoint of the window. These dates are referenced as calendar dates in the main text and date indices (number of days before/after January 1, 1950) in the supplement for convenience. In practice, for the analysis presented in this work, only the year collected is relevant.
For the regional analysis, the latitude and longitude of each city of origin or a representative city for each country/administrative region of origin was identified from simplemaps (https://simplemaps.com/data/world-cities) (7). This information was used to calculate the distance on the globe between pairs of sequencing locations (the great circle distance, not respecting geographic or political boundaries).

Tree Construction

With the exception of SARS-CoV-2 and H3N2, tree topology was optimized using IQ-TREE (8) with the evolutionary model fixed to GTR+F+G4 and the minimum branch length decreased from the default 10e-6 to 10e-7 (options: -m GTR+F+G4 -st DNA -blmin 0.0000001). For SARS-CoV-2, the tree was drawn from the global topology previously described (4) corresponding to the selected subalignment. For H3N2, trees for both subalignments (evenly sampled and maximum diversity) were constructed as described using IQ-TREE. The global H3N2 tree was approximated using FastTree(9) specifying GTR; a 4 category gamma distribution; no support values; and using the previously-constructed maximum diversity subtree as a constraint (compiled at double precision, options: -nt -gtr -gamma -cat 4 -nosupport -constraints).
Trees were rooted according to the position of an outgroup selected from representative sequences of the closest relative species whenever the percent identity of the largest protein or polyprotein was at least 30%. In three cases, the outgroup did not meet these criteria. We applied midpoint tooting for HDV andHRSV) and rooted at the oldest sample for H3N2.

Manual Parsing of Trees

Viral lineages were manually selected representing viral sero- or genotypes, known geographic subtypes, and large, monophyletic clades. No manual lineages were selected for EVB and Rhinovirus A (RVA). Sequences of each manual lineage ("ML") are listed in Supplementary Data, Table S6.

Parsing Trees into Correlated Subsets Defined by Sequencing Date and Root Distance ("genealogical lineages")

In addition to these manually established subtypes, we sought to identify individual "outbreaks" within the trees with a well defined evolutionary "trajectory" composed of a monophyletic clade such that within this clade, the distance to the tree root is well correlated with the date of isolation. Moving from root to tip, at each node of the global topology, we calculated the Pearson correlation coefficient between the root distance and the isolation date for all sequences descendent from that node. For a predefined threshold, C, if the correlation was greater than C, this clade was added to the ensemble of correlated clades and the search was stopped at that node. If the correlation was below C, the search would continue until the descendent clades contained fewer than 15 descendent sequences. Such clades with fewer than 15 sequences were not considered further.
The threshold, C, was then determined for each tree to be either 0.8 or the largest value such that 30% of all sequences with known isolation dates were included in a correlated clade, whichever was smaller with the exception of SARS-CoV-2. The threshold for SARS-CoV-2 was set to 0.75 as between 0.75 and 0.8 this parsing scheme dramatically differed from returning a single, inclusive correlated clade to returning over 40 small clades. At 0.75, all but one sequence was included, and consequently we considered all sequences in SARS-CoV-2 to belong to a single correlated clade. C was found to be less than 0.2 for two trees (HDV/0.18 and RVA/0.16); less than 0.65 for five more (Ebola/0.59, EVB/0.41, HCV/0.63, PeVA/0.39 and TBEV/0.49); and 0.8 for all others (with the exception of SARS-CoV-2). H3N2 sequences display a very high root distance/sequencing date correlation (Fig. S7) and nearly all H3N2 sequences formed a single correlated clade even at a threshold value near 1 with the method described above; however, due to the ladder-like tree structure with relatively fewer sequences near the root, parsing near the root with this method is sensitive to outliers and rooting. Consequently, we considered all sequences in H3N2 to belong to a single correlated clade. Note as discussed in the main text, we refer to these correlated clades as "genealogical lineages" or GL.
Subtrees corresponding to the subalignments described above for H3N2 and EVA were additionally considered as well as two subtrees for H3N2 representing "newer" and "older" sequences (H3N2n/H3N2o) roughly determined to be sequenced after/before 2010 respectively.
Sequences of each correlated/ genealogical lineage ("GL") are listed in Supplementary Data, Table S6 and sequences for H3N2n and H3N2o are in Supplementary Data, Table S7.

Construction of Date-Constrained Genealogical Trees

All trees, global topologies for each alignment as well as the selected subtrees, with branch lengths corresponding to the number of nucleotide substitutions per site, were paired with the known dates of isolation to construct date-constrained, genealogical trees using least-square dating (with software LSD2) (10) associated with a single mutation rate for each tree/subtree and, consequently, a predicted date for the LCA of each tree/subtree (Supplementary Data, Table S8). When not known, the isolation dates were estimated and unconstrained so that, in some trees, select sequences are predicted to have been isolated in the future.

Many of these clades are far from the tree root with few sequences interspersed between the clade root and the tree root. The mutation rate along these long, deep connecting branches may differ significantly from that estimated over the whole tree or among the GLs. If present, such a rate mismatch would substantially change the predicted date for the LCA. This date, as well as the predicted dates of other deep nodes, is used to estimate the effective population size. We are primarily interested in establishing a lower bound, but not necessarily an upper bound, for the effective population size (see below) and given the sparsity of these branches, a partitioned evolutionary model is unlikely to yield statistically significant results. Alternatively, we constructed truncated global genealogical trees, or “grafted trees”, as follows. First the deepest root (oldest LCA) of any GL was identified. This was taken to be the root for the grafted tree. Next, the remaining GLs were connected to this (now multifurcated) root by branches reflecting the difference between the LCA of each clade and the root. These grafted trees represent an upper bound on the mutation rates over these branches and the effective population size estimated over these grafted trees represents the lower bound for the effective population size of each virus respecting the genealogical trees estimated over each GL.

Given the original trees as well as the date-constrained, genealogical trees, we sought to construct a measure representing the rate at which older GLs were displaced by more recent GLs (for those trees with at least 2 GLs identified). This is related to the rate at which clades go extinct. We considered the Shannon entropy of the clade distribution calculated over sliding windows containing the nearest 5% of all isolates (including redundant sequences and not limited to tree leaves) based on the known or estimated date of isolation S_t^j OR distance to the tree root S_d^j (see main text). Dates/root distances were sampled from that corresponding to the earliest or deepest isolate in any GL to the latest or shallowest respectively. The distance to the root was linearly scaled to represent the date of isolation corresponding to each sequence in an alternative genealogical tree where the date of isolation for each sequence exactly corresponds to the distance to the root for that sequence (mapped to the established date range).

The entropy is reported in units of base *N*, the number of GLs in the tree so that the maximum value is 1 and the minimum value is 0 for all trees. A mean entropy near 0 represents a genealogical tree composed of clades which quickly displace one another. A mean entropy near 1 represents a genealogical tree where all clades are equally distributed at every time point. We observe $\langle S_t^j \rangle_j > \langle S_d^j \rangle_j$ in all but one case (HDV) indicating that the entropy is larger than that expected from an analysis of the tree structure with no known dates of isolation (see Fig. 3D).

Reconstruction of Ancestral Genome Sequences

Fitch Traceback (11) was used to estimate ancestral states. Briefly, character sets were constructed moving from leaf to root. Each node was assigned the intersection of the descendant character sets if not empty and the union otherwise. Moving from root to leaf, nodes with more than one character in their set were assigned the consensus character if present in their set or a randomly chosen representative character otherwise. Substitutions between states were identified and placed in the middle of the branch bridging the pair of nodes.

Mutational Signatures

In an effort to distinguish intra-GL and inter-GL mutational signatures, three classes of amino acid sites were identified on the basis of the nonsynonymous mutations within each site. Redundant sequences were not evaluated in this analysis. 1) Multiple, deep (MD) substitutions are “lineage defining”, being conserved in at least 90% of the samples within at least two GLs, but represented

by different amino acid residues in each of these GLs. For example, consider the third amino acid of the CHIKV ORF gp1. 97% of the sequences in GL 1 contain a Serine in that site while 96% of the sequences in GL2 contain a Proline in that site.2) Multiple, shallow sites. Consider a nonsynonymous mutation in site *M* which appears independently *N* times across all GLs combined (but may appear only once within any GL). If *N* is no less than the threshold defined to be the 90th percentile of the distribution over all *N* or 2 (whichever is larger), site *M* is included in this class of repeat, shallow sites. 3) All shallow sites bearing at least one nonsynonymous mutation which occurs within a GL. Only viruses with at least 2 GLs were included in this analysis. We then computed the site density of each class over a sliding window of 101 amino acid sites respecting mature peptide boundaries. The site densities across the genomes are displayed (normalized with respect to the mean site density for each class over the entire genome) in Supplemental Figures S18-24.

Computation of *dN/dS*

For each mature protein sequence within each alignment, 10 reduced alignments were constructed as follows. Sequences were iteratively included in each reduced alignment to maximize diversity by maximizing the minimum hamming distance to the ensemble at each step. The first 10 sequences are conserved across every alignment and the remaining 40 are unique to each alignment. The reference sequence, was additionally added to each reduced alignment and PAML(12) was used to estimate tN, tS, *dN/dS*, N, S, and N/S for each segment and every reduced alignment. For sub-lineages (manually selected and GL) containing less than 410 sequences, the maximal number of reduced alignments was constructed. The reference sequence was not added to reduced alignments of sub-lineages.
Runs with total tree lengths above 50 (with Branch length defined as number of nucleotide substitutions per codon) were removed (resulting in fewer than 10 independent estimates for some viruses). These cases most likely represent ML estimates which did not converge. Median *dN/dS* is calculated for each peptide as well as mean *dN/dS* over all proteins (normalized to their length). Whenever saturation of *dS* (>1.5) is observed for any reduced alignment, the corresponding peptides are highlighted in the respective figures. No *dN/dS* is given for the following peptides: EVCr_sub3 2B and VPg, OHVA_sub1 A1pp, OHVA_sub3 Viral helicase and TBEV_sub2 ancC. In these cases, the sub lineages contained fewer than 90 samples resulting in only one reduced alignment showing an unreliable total branch length above 50 nucleotide substitutions per codon. Mean *dN/dS* ratios for all peptides of all lineages can be found in Supplementary Data, Table S9.

Estimation of the Total and Effective Population Size

The effective population size *Ne* and the ratio of the census population size *N* over *Ne* was estimated as previously described (13). In brief, genealogical trees were analyzed with the PACT package (http://www.trevorbedford.com/pact). Here, coalescence rate (Cr) is defined by the number of possible coalescent events (equal to the number of internal branches) divided by the opportunity for a coalescent event in a given fixed window of time.

The opportunity is defined as

$$opportunity = \sum_i \frac{kn(n-1)}{2}$$

With *k* being the length of the interval, *n* the number of concurrent lineages and *i* the discrete set of intervals. In our case, each tree is sliced into 1000 equal parts (*i*) from root to most distant tip with the tree specific length of the interval *k* in years. Given

$$Ne * t \sim 1/Cr$$

where *t* is the viral generation time, one can estimate *Ne* in generations which is equal to the number of individuals assuming the Fisher-Wright model (see, for example, (14)). Generation

time *t* was estimated for each virus based on available epidemiological data. To retrieve *N/Ne* ratios, yearly cases were estimated for each virus and N was calculated as

$$N = D * t$$

with *D* being estimated yearly cases. *t* and *N* estimations can be found in Supplementary Data, Table S10, including references.

Estimation of diversity and TMRCA over time in skyline plots

Viral diversity (average time of any pair of leaves at a given timepoint to find their LCA) and average time to most recent common ancestor (TMRCA) over time were calculated with the PACT package(http://www.trevorbedford.com/pact). Within PACT, step size was set to 2 years to avoid windows with only one branch for which no opportunity could be calculated (see above). The analysis was repeated four times with a 0.25-year shift for the 2-year window to allow optimal resolution. At each timepoint, a cross-section of the tree is taken. Branches cut at this section are treated as terminal leaves. Then, diversity and mean TMRCA are calculated for this timepoint. Skyline plots for main virus populations, estimations from grafted trees, and all GLs can be found in the Supplementary Data.

Simulating trees with respect to selection strength and sampling density

In order to demonstrate the potential equivalency between the impacts of selection strength and sampling density on effective population size, we simulated an ensemble of trees as follows. Beginning with a rooted, bifurcated tree consisting of 2 leaves with uniformly distributed random branch lengths (between 0 and 1), the tree was extended by adding a multifurcated node at the "end" of a leaf so that the terminal branch length became the branch leading to the added multifurcation. The branch lengths of all leaves within the multifurcation were also random and uniformly distributed between 0 and 1. This process was continued for 500 iterations. At each iteration up to 10 leaves were selected for extension. To represent increased sampling, the multifurcation multiplicity was varied from 2 to 6. To represent increased selection strength, leaves were selected for extension only if they were positioned beyond a given threshold distance to the root of the tree. This threshold was set to be the 0th, 25th, 50th, 75th, or 95th percentile of the root distance distribution for the entire tree at that iteration. Note that in practice, multifurcations were represented as bifurcating subtrees with arbitrary topology and arbitrarily short interior branch lengths.

Explicit demonstration of non-neutrality, comparison with Yule-Harding model

For each virus, the largest GL (or the entire genealogical tree in the absence of identified GLs) was selected. The minimum, mean, and maximum branch length (in units of time) within each GL was calculated and a Yule-Harding tree, which reasonably approximates neutral evolution (15, 16) with 100k leaves was simulated (iqtree (8) command: iqtree -r 100000 -rlen {min} {mean} {max}). These branch length statistics were computed within a GL rather than the entire genealogical tree to exclude branches connecting GLs which, generally, represent periods over which sequencing was unavailable. Each simulated tree was then rooted at the deepest internal node and the maximum distance to this root for any leaf was identified. The real genealogical trees of the viruses studied generally lack sequences near the root due, principally, to the fact that the MRCAs are predicted to have circulated much earlier than the invention of sequencing technologies. Accordingly, the simulated trees were sub-sampled to reflect this structure. A threshold root-distance was computed such that the ratio between this window of time (from threshold to tree tips) and the maximum distance to the root for each simulated tree, is the same as the ratio between the period over which sequencing technologies were available (approximately 75 years from the time of writing, 2022) and the period from the present to the predicted date of the MRCA for each real genealogical tree (note that for EVB, the largest GL is used in place of the whole phylogeny for which the calculation of a single mutation rate diverged):

$$threshold \equiv max(root\ distance) - \frac{75}{2022 - MRCA} max(root\ distance)$$

Simulated trees were then subsampled, removing sequences below this threshold; however, this threshold is very high for viruses with ancient MRCAs leaving too few remaining sequences for statistical analysis. In these cases, the threshold was reduced (alternatively simulated trees with more than 100k leaves could be generated but this becomes computationally impractical and is unlikely to qualitatively affect the results presented).
Specifically, simulated trees with fewer than 100 sequences above this threshold were pruned to select the 100 leaves with the greatest distance to the root. Simulated trees with more than 100 leaves above this threshold but fewer than the number of sequences in the real phylogeny were pruned above the threshold. Simulated trees with more leaves above the threshold than sequences in the real phylogeny were pruned above the threshold and then randomly sampled to contain the same number of sequences. If the final, pruned, simulated tree contained fewer sequences than the real phylogeny, the real phylogeny was then subsampled to maximize Hamming-distance diversity among the remaining sequences (see section "Selecting subalignments for EVA and H3N2") to match the number of sequences within the real and simulated trees. Thus, this procedure results in simulated genealogical trees for each virus, approximating the expected result of neutral evolution from the MRCA to the present and sampled to respect the period of time over which sequencing technologies were available (as well as, subsampled where appropriate, real genealogical trees with the same number of leaves) all are available in the Supplementary Data. While it is possible to analyze these trees in PACT as was done above, PACT estimations are most robust within the timeframe of sampling (oldest to youngest leaf, where coalescence events are frequent). Here we are interested in studying precisely the opposite portion of each tree, between the MRCA and the oldest sample. Further note that for the analysis conducted in this section, the absolute branch lengths within each tree are irrelevant and the branch lengths of the simulated trees (available in the Supplementary Data) must be rescaled if absolute comparisons are desired.
For all trees, real and simulated, the number of parallel branches from the root to the tree tips was computed. In order to probe the coalescence rate among, rather than within, extant lineages, the date associated with the deepest leaves (specifically the 5th percentile of the root distance distribution for the leaves) was determined and the period from the root to this point was further examined. The number of branches, B, from the deepest leaves back towards the root was then fit to a single-parameter, *a*, power law expression (based on expectations of a power-law distribution of node descendants (17)) where time, *t*, is indexed in reverse; B_0 is the maximum number of parallel branches (over this period); and B_1 is the mean number of parallel branches over the final 5th percent of the window nearest to the root (being binary trees, this value is usually 2):

$$B = a(1 + a)(B_0 - B_1)(t + a)^{-1} + (1 + a)B_1 - aB_0$$

(see Figures S31-35). The derivative of each curve (see Figures S36-S40) as well as the log-fold difference between the derivative of the simulation and the real genealogical tree for each virus was computed (Figure S41).

Metadata

Metadata including the sequence ID, host, date of isolation, location of isolation, and subtype for all sequences is available in Supplementary Data, Table S11.

Additional statistics

Arithmetic and geometric mean was calculated as indicated in Table S12 and presented with SD or log10 SD, respectively. Linear regression presented in Fig. S28 was calculated with python package scipy.

List of abbreviations

AS: All, shallow
BCoV1: Betacoronavirus 1
CHIKV: Chikungunya virus
DENV: Dengue virus
dN/dS: ratio of non-synonymous to synonymous substitution rates
DUF: Domain of unknown function
Ebola: Zaire ebolavirus
EVA: Enterovirus A
EVAd: Enterovirus A 'diverse'
EVAe: Enterovirus A 'even'
EVB: Enterovirus B
EVC: Enterovirus C
EVCe: Enterovirus C 'every' sample
EVCr: Enterovirus C 'reduced' samples
EVD: Enterovirus D
GL: Genealogical lineage
H3N2d: H3N2 'divers'
H3N2e: H3N2 'even'
HBV: Hepatitis B virus
HCV: Hepatitis C virus
HCVe: Hepatitis C virus 'every' sample
HCVr: Hepatitis C virus 'reduced' samples
HDV: Hepatitis D virus
HMPV: Human metapneumovirus
HRSV: Human respiratory syncytial virus
HRV3: Human respirovirus 3
H3N2: Influenza A Virus H3N2
LCA: Last common ancestor
LHDAg: Large human delta antigen
LSD: least-square distance
MD: Multiple, deep
MERS: Middle East respiratory syndrome-related coronavirus
ML: Manual lineage
MMV: Measles morbillivirus
MRCA: Most recent common ancestor
MRV: Mumps rubulavirus
MS: Multiple, shallow
N: Census population size
Ne: Effective population size
Norwalk: Norwalk virus
NS: Non-structural protein
NSP: Non-structural protein
OHVA: Orthohepevirus A
PeVA: Parechovirus A
RVA: Rhinovirus A
SARS-CoV-2: Severe acute respiratory syndrome-related coronavirus 2
SV: Sapporo virus
TMRCA: Time to most recent common ancestor
YFV: Yellow fever virus
ZIKV: Zika virus

Declarations

Ethics approval and consent to participate

Not applicable.

Consent for publication

Not applicable.

Data Availability

The datasets generated and/or analyzed during the current study are available as supplementary data at Zenodo, https://doi.org/10.5281/zenodo.5711959, as well as through FTP, https://ftp.ncbi.nih.gov/pub/wolf/_suppl/virNiches/. Original virus sequences are publicly available for all viruses except SARS-CoV-2 at NCBI virus (1). SARS-CoV-2 sequences are available at GISAID (3).

Competing interests

The authors declare that they have no competing interests.

Funding

NDR, YIW PM, and EVK are supported by the Intramural Research Program of the National Institutes of Health (National Library of Medicine).

Authors' contributions

PM, NDR, and GF collected data; PM, NDR, YIW, GF, FZ, and EVK analyzed data; PM, NDR, and EVK wrote the manuscript that was edited and approved by all authors.

Acknowledgements

The authors thank Koonin group members for helpful discussions.

Supplementary Data

Tables

Table S1: Virus characteristics including abbreviation, family, order, tax id, sequence length threshold (for nearly complete genomes), vaccination status, mode of transmission, circulation (human or human/zoonotic), available treatment, and disease progression (acute or chronic).
Table S2: Sequence IDs of outgroup constituents.
Table S3: GISAID acknowledgements for the SARS-CoV-2 sequences used in this study.
Table S4: Summary of manual sequence curation indicating lab- or vaccine-related keywords used to prune sequences.
Table S5: Sequence IDs for EVA and H3N2 e10, e100, d10, and d100 subtrees.
Table S6: Sequence IDs for ML and GL lineages.
Table S7: Sequence IDs for the H3N2 "new" and "old" subtrees.
Table S8: Mutation rates (in units of nucleotide substitutions per site per year) and estimated dates for the last common ancestor of all tree/subtrees. Negative dates represent years prior to 0 CE.
Table S9: Mean dN/dS values.
Table S10: Yearly viral cases and generation time estimations (with references).
Table S11: Metadata including the sequence ID, host, date of isolation, location of isolation, and subtype.

Table S12: Overview of key parameters including number of GLs N, Ne, and MRCA for each virus species as well as the mean over all species.

Directories

Alignments ORFeome alignments (excluding stop codons) with the exception of SARS-CoV-2.
Correlated Subtrees Subtrees representing all correlated-clades (genealogical lineages; GL) in Newick format.
Diversity TMRCA and Skyline Plots .png files.
e/d/n/o Subtrees EVA and H3N2 subtrees, evenly and diversely sampled as well as "early" and "late" subtrees for H3N2.
Genealogical Trees Including global topologies (.main), subtrees, and grafted trees (.grafted).
Maximally Diverse Subtrees Genealogical trees sampled to contain the same number of leaves as each respective tree in the Simulated Trees directory.
ORF References The first and last nucleotide of each ORF in the reference sequence.
Redundancy Tables List of all redundant isolates and the corresponding representative in the ORFeome-unique alignment.
Reference Sequences Genbank page for all reference sequences.
Rooted Trees Global topologies for each virus.
Simulated Trees Neutral models, based on Yule-Harding, for each virus.
Ultrametric Trees For each global topology.

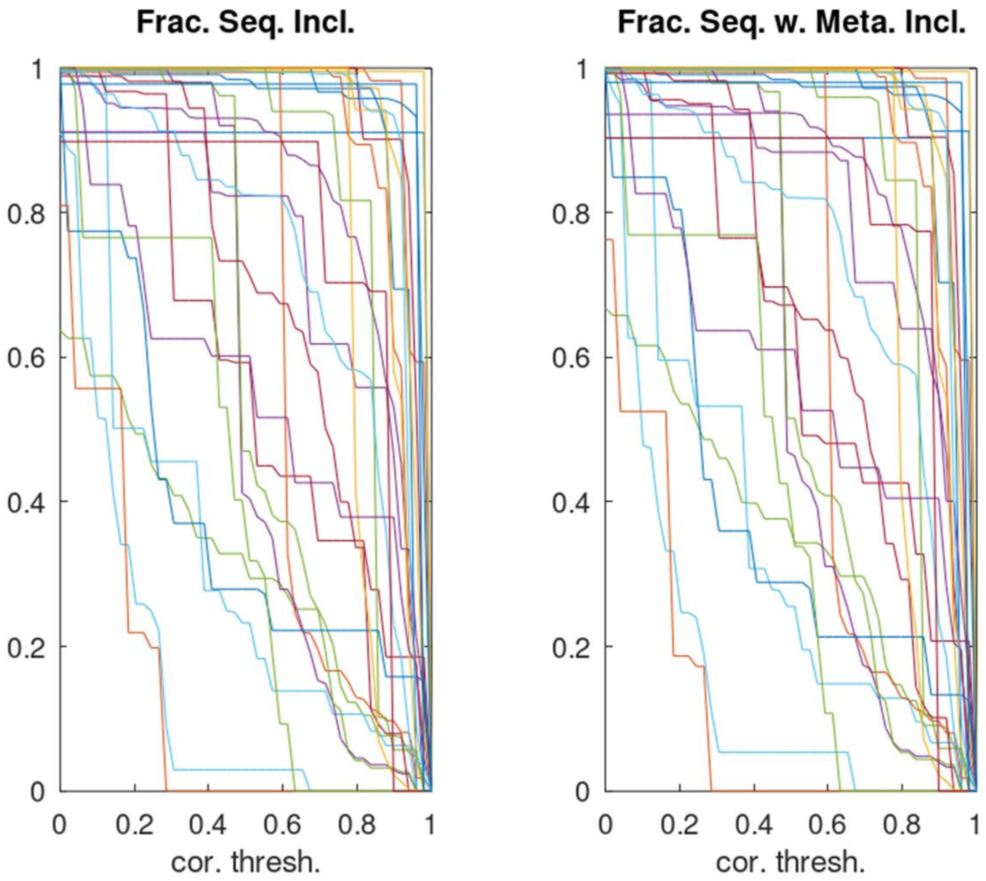

Fig. S1. The fraction of sequences included in any correlated-clade (genealogical lineage) as a function of the Pearson correlation coefficient threshold. Left. All sequences. Right. Sequences with metadata.

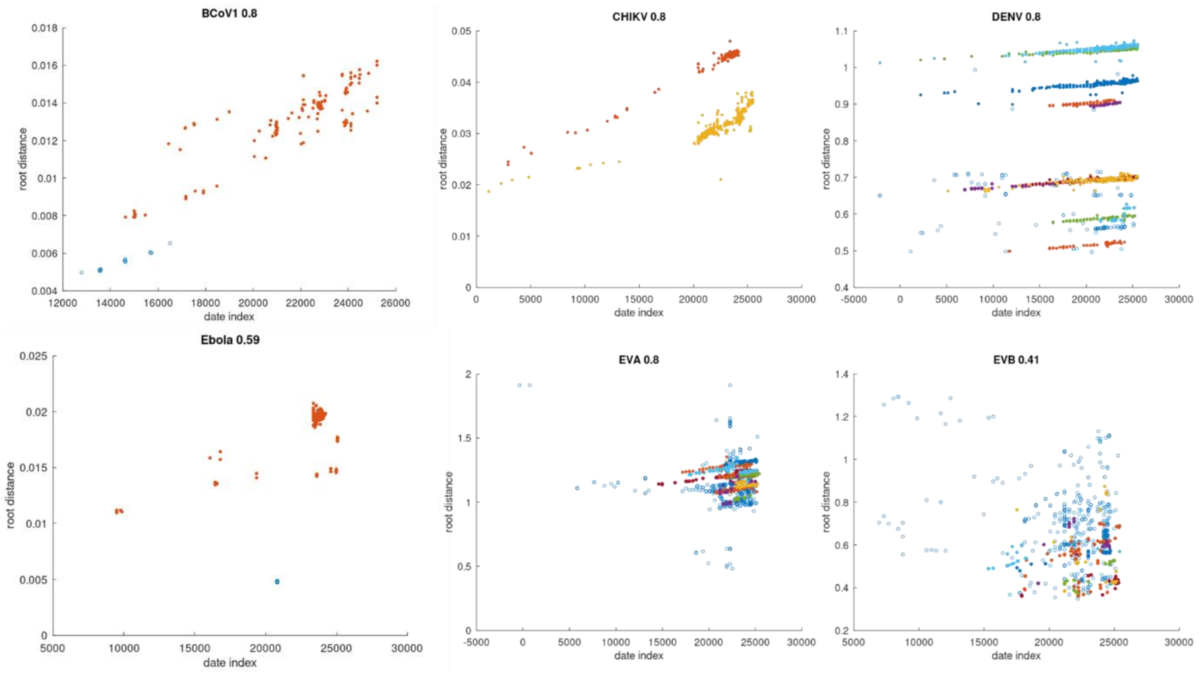

Fig. S2. The distance to the tree root vs. the date of isolation. Each point represents a leaf in the tree. Filled circles represent leaves included in a correlated-clade and are colored accordingly. Open circles represent leaves which are not included in any correlate clade.

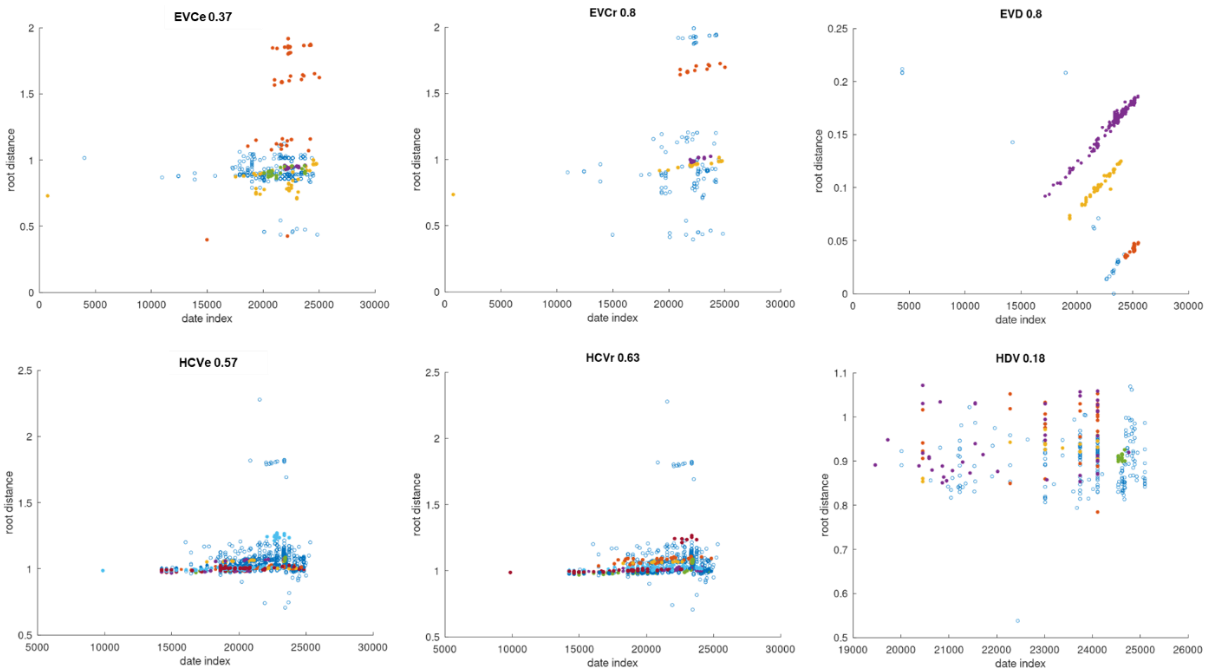

Fig. S3. The distance to the tree root vs. the date of isolation. Each point represents a leaf in the tree. Filled circles represent leaves included in a correlated-clade and are colored accordingly. Open circles represent leaves which are not included in any correlate clade.

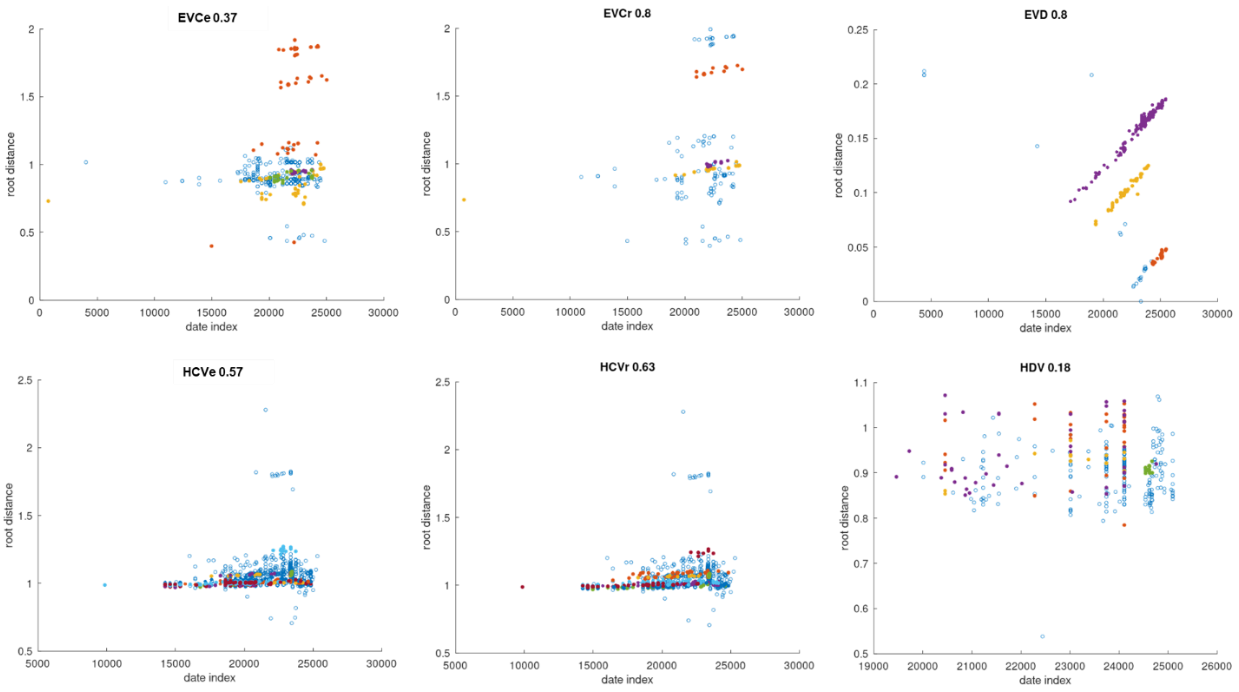

Fig. S4. The distance to the tree root vs. the date of isolation. Each point represents a leaf in the tree. Filled circles represent leaves included in a correlated-clade and are colored accordingly. Open circles represent leaves which are not included in any correlate clade.

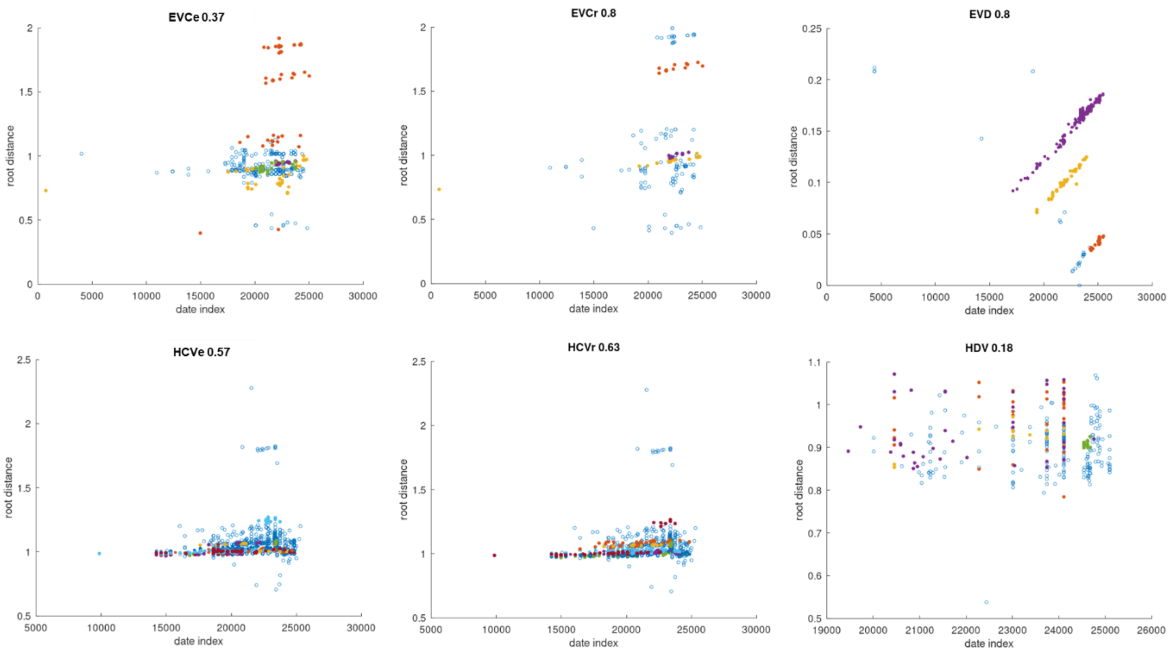

Fig. S5. The distance to the tree root vs. the date of isolation. Each point represents a leaf in the tree. Filled circles represent leaves included in a correlated-clade and are colored accordingly. Open circles represent leaves which are not included in any correlate clade.

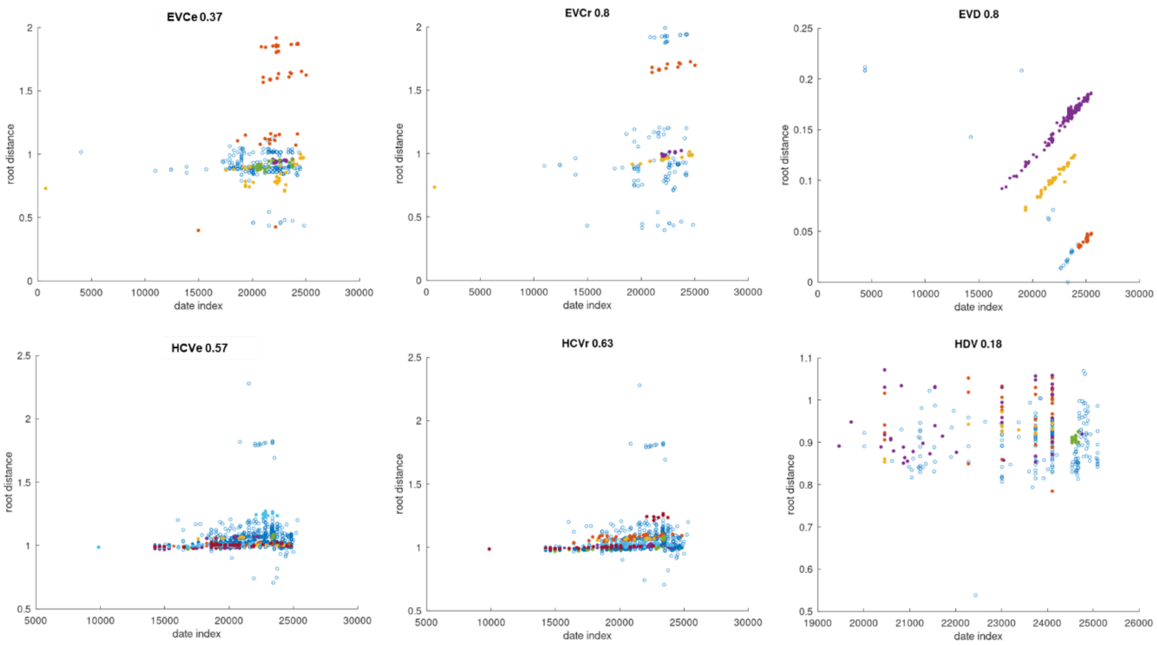

Fig. S6. The distance to the tree root vs. the date of isolation. Each point represents a leaf in the tree. Filled circles represent leaves included in a correlated-clade and are colored accordingly. Open circles represent leaves which are not included in any correlate clade.

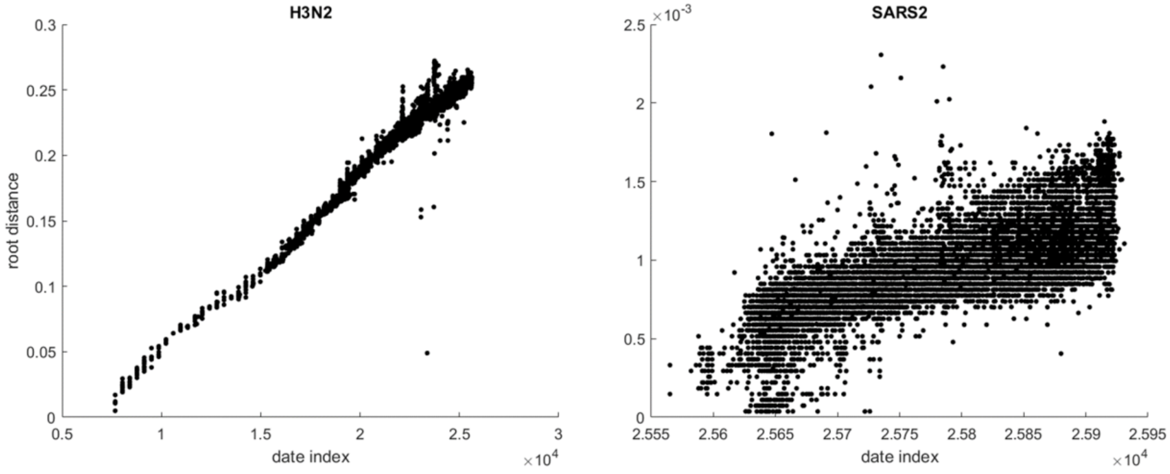

Fig. S7. The distance to the tree root vs. the date of isolation. Each point represents a leaf in the tree. Filled circles represent leaves included in a correlated-clade and are colored accordingly. Open circles represent leaves which are not included in any correlate clade. For SARS-CoV-2 and H3N2 the entire tree was considered to constitute a single, correlated-clade.

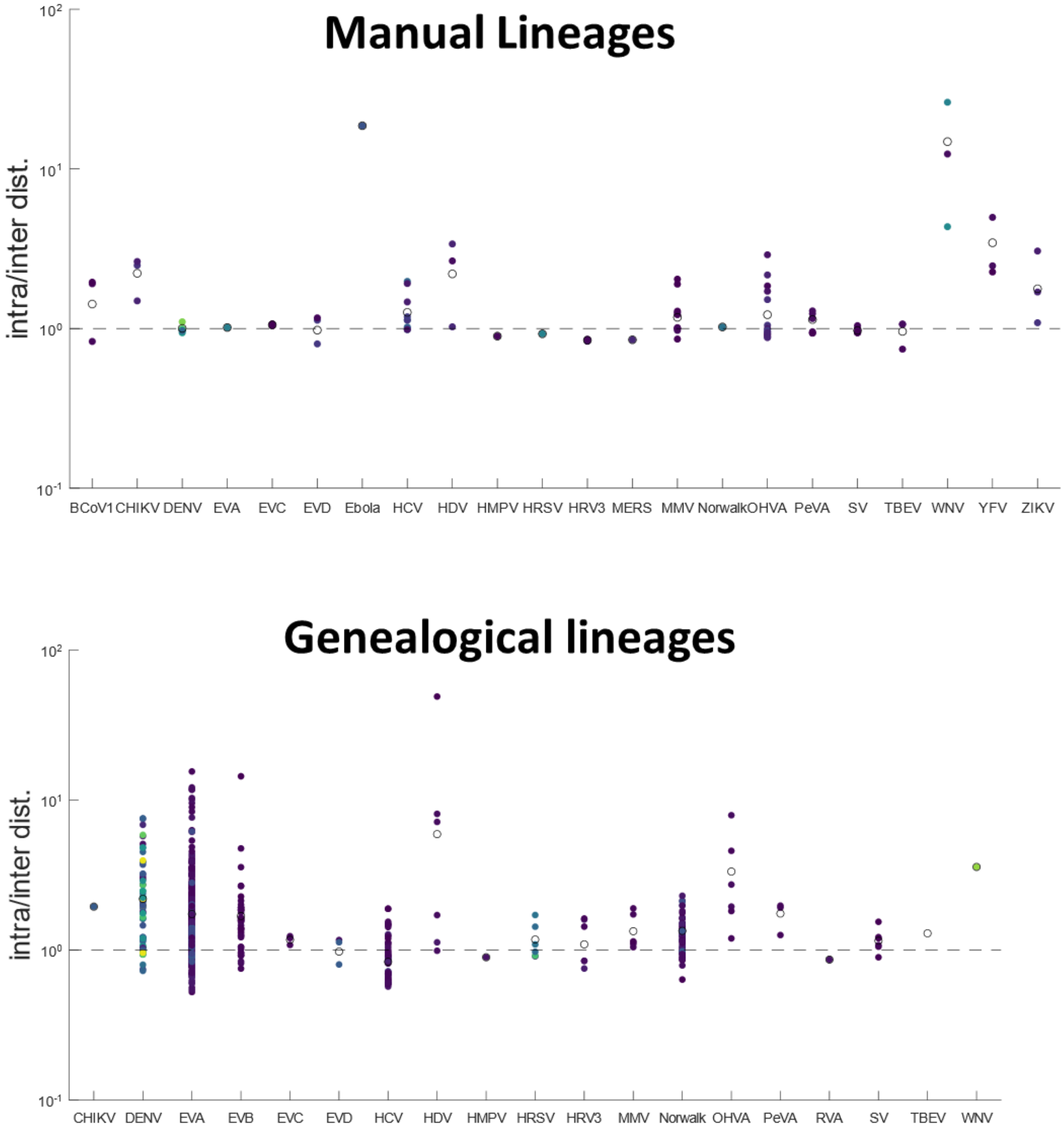

Fig. S8. The ratio of the mean inter- vs. intra-lineage map distance for all pairs of lineages for manually selected lineages on top and genealogical lineages (GL) below. The mean inter-lineage map distance is the mean great circle distance between sequencing locations taken over all pairs of isolates spanning the pair of lineages. The mean intra-lineage map distance is taken over all pairs of isolates within each lineage and averaged over the two mean values for each lineage. Filled circles represent pairs of lineages where the colors represents the number of isolates within the pair. Open circles represent the mean ratio weighted by the number of isolates in each pair.

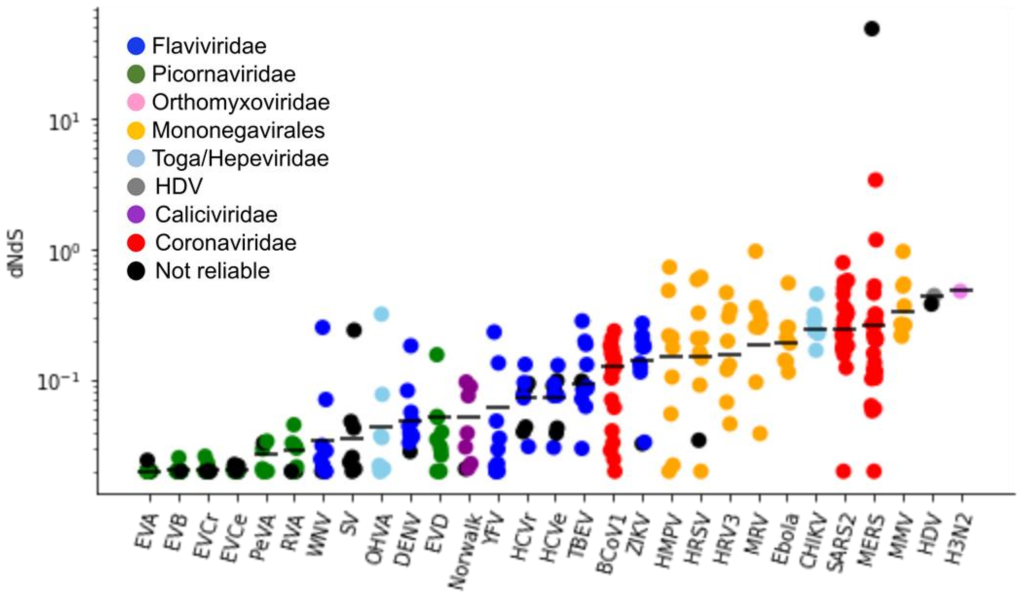

Fig. S9. *dN/dS* ratios were analysed for each protein of each virus and are represented by a single dot. Black line represents mean over all proteins (normalized to length). Black dots represent proteins with non-reliable *dN/dS* due to saturation of synonymous substitutions.

.

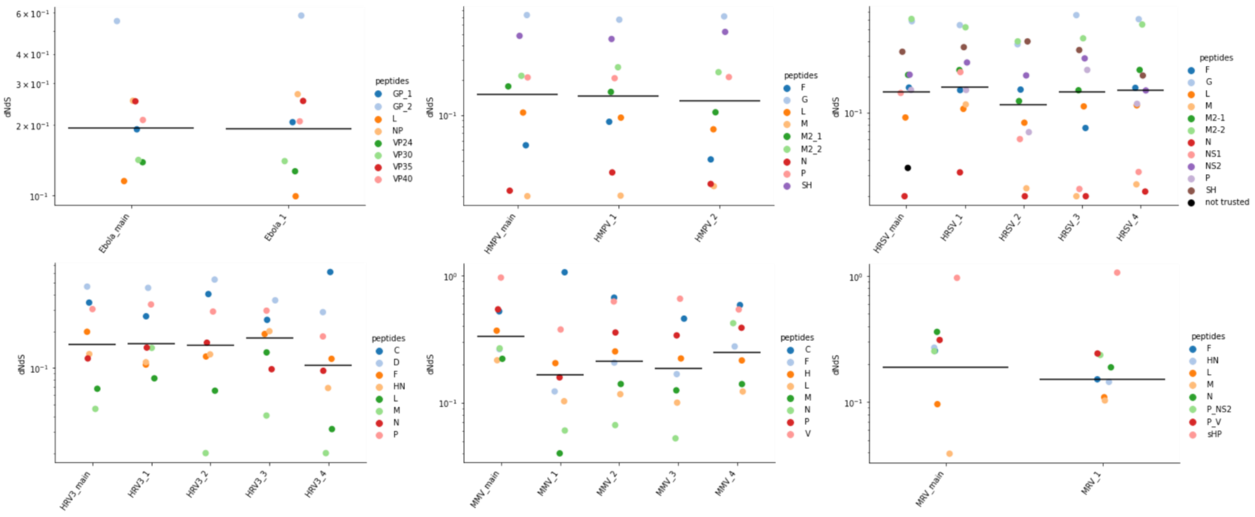

Fig. S10. *dN/dS* ratios retrieved from trees covering the whole population (corresponding to Figure S9) and trees representing genealogical lineages (GL). Each protein is represented by a single dot, black line represents mean over all proteins. Black coloured dots represent proteins with non-reliable *dN/dS* due to saturation of synonymous substitutions.

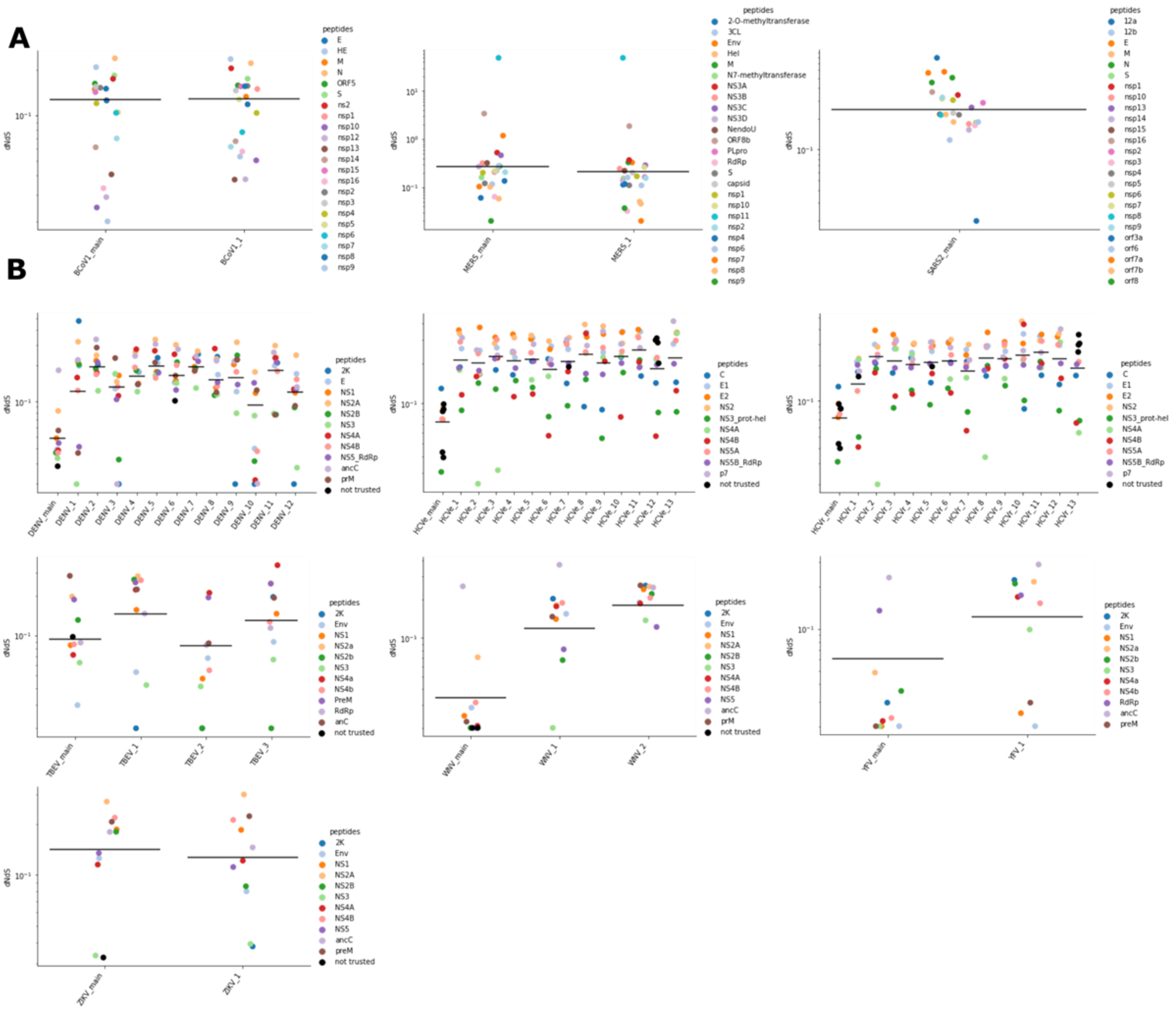

Fig. S11. *dN/dS* ratios retrieved from trees covering the whole population (corresponding to Figure S9) and trees representing genealogical lineages (GL). Each protein is represented by a single dot, black line represents mean over all proteins. Black coloured dots represent proteins with non-reliable *dN/dS* due to saturation of synonymous substitutions.

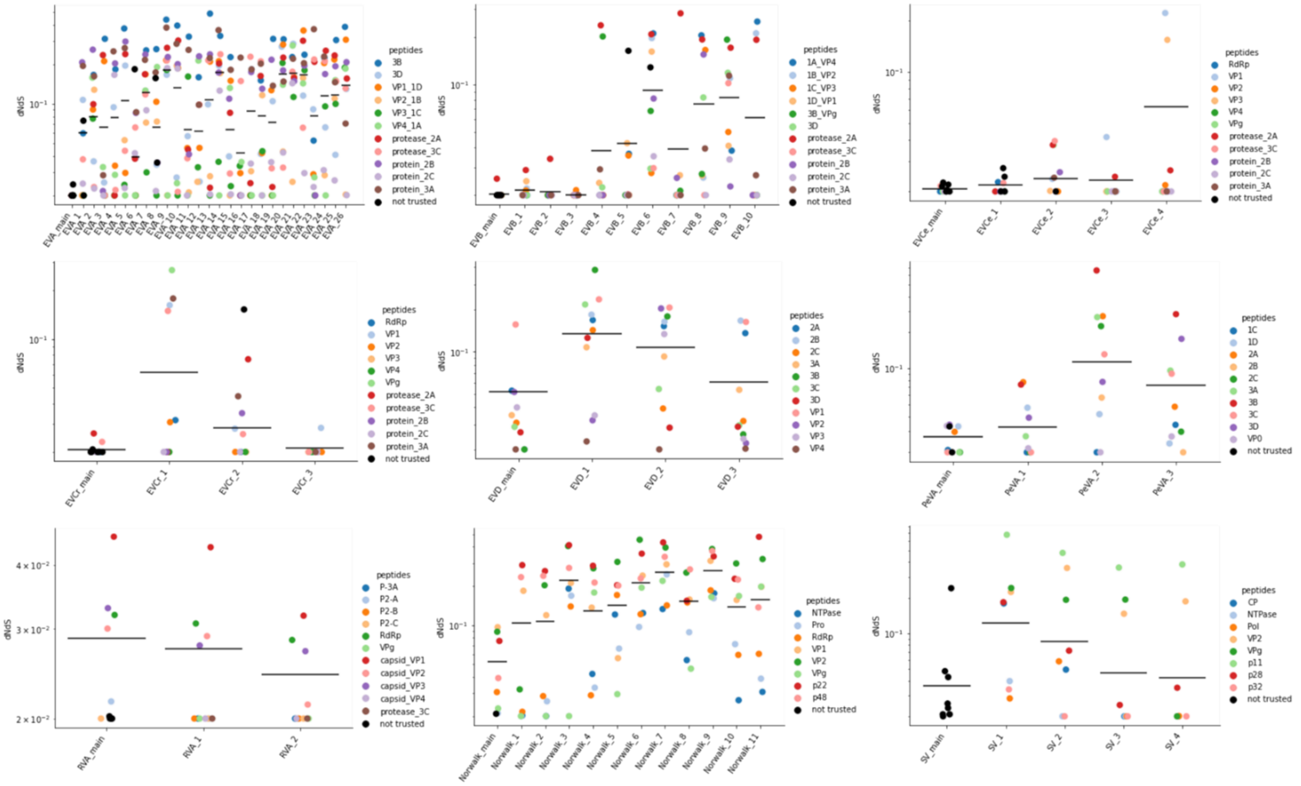

Fig. S12. *dN/dS* ratios retrieved from trees covering the whole population (corresponding to Figure S9) and trees representing genealogical lineages (GL). Each protein is represented by a single dot, black line represents mean over all proteins. Black coloured dots represent proteins with non-reliable *dN/dS* due to saturation of synonymous substitutions.

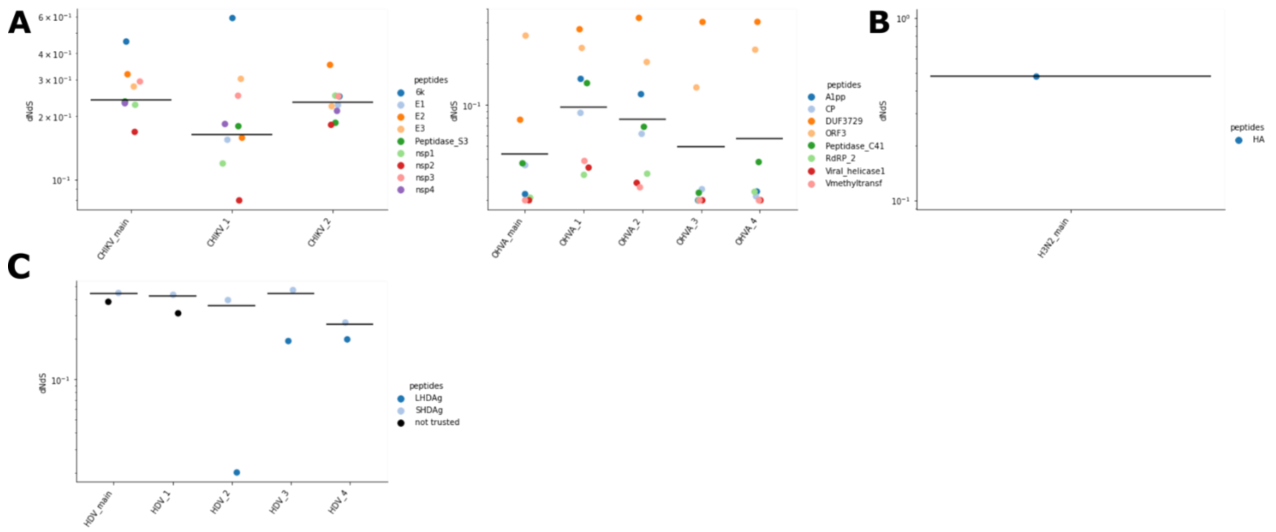

Fig. S13. *dN/dS* ratios retrieved from trees covering the whole population (corresponding to Figure S9) and trees representing genealogical lineages (GL). Each protein is represented by a single dot, black line represents mean over all proteins. Black coloured dots represent proteins with non-reliable *dN/dS* due to saturation of synonymous substitutions.

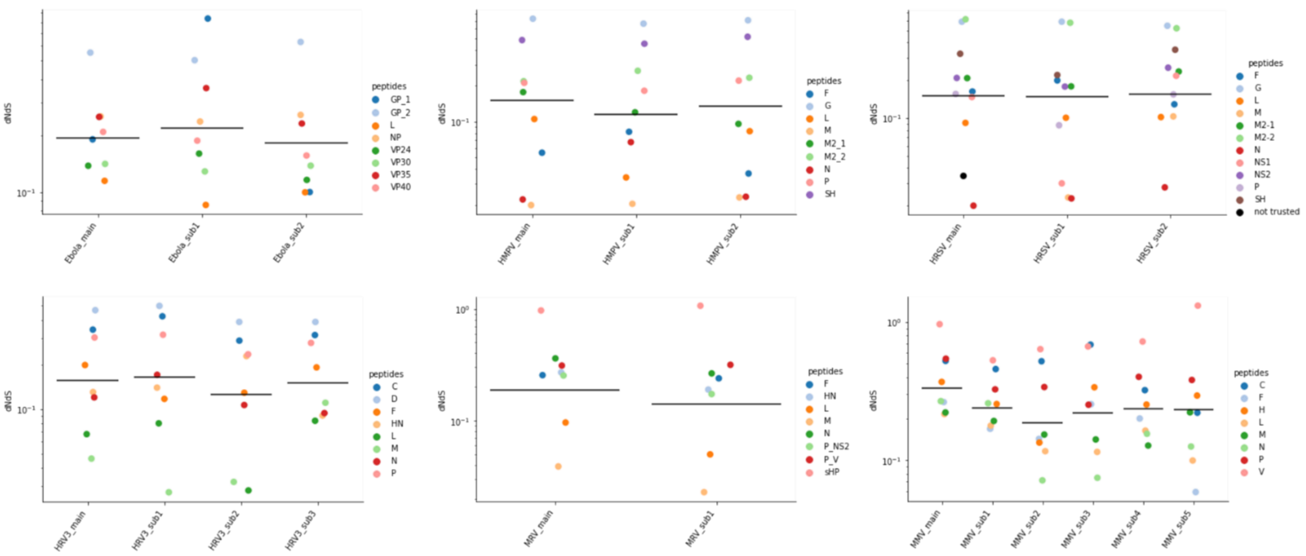

Fig. S14. *dN/dS* ratios retrieved from trees covering the whole population (corresponding to Figure S9) and trees representing manually selected lineages (correlating to viral sero- or genotypes). Each protein is represented by a single dot, black line represents mean over all proteins. Black coloured dots represent proteins with non-reliable *dN/dS* due to saturation of synonymous substitutions.

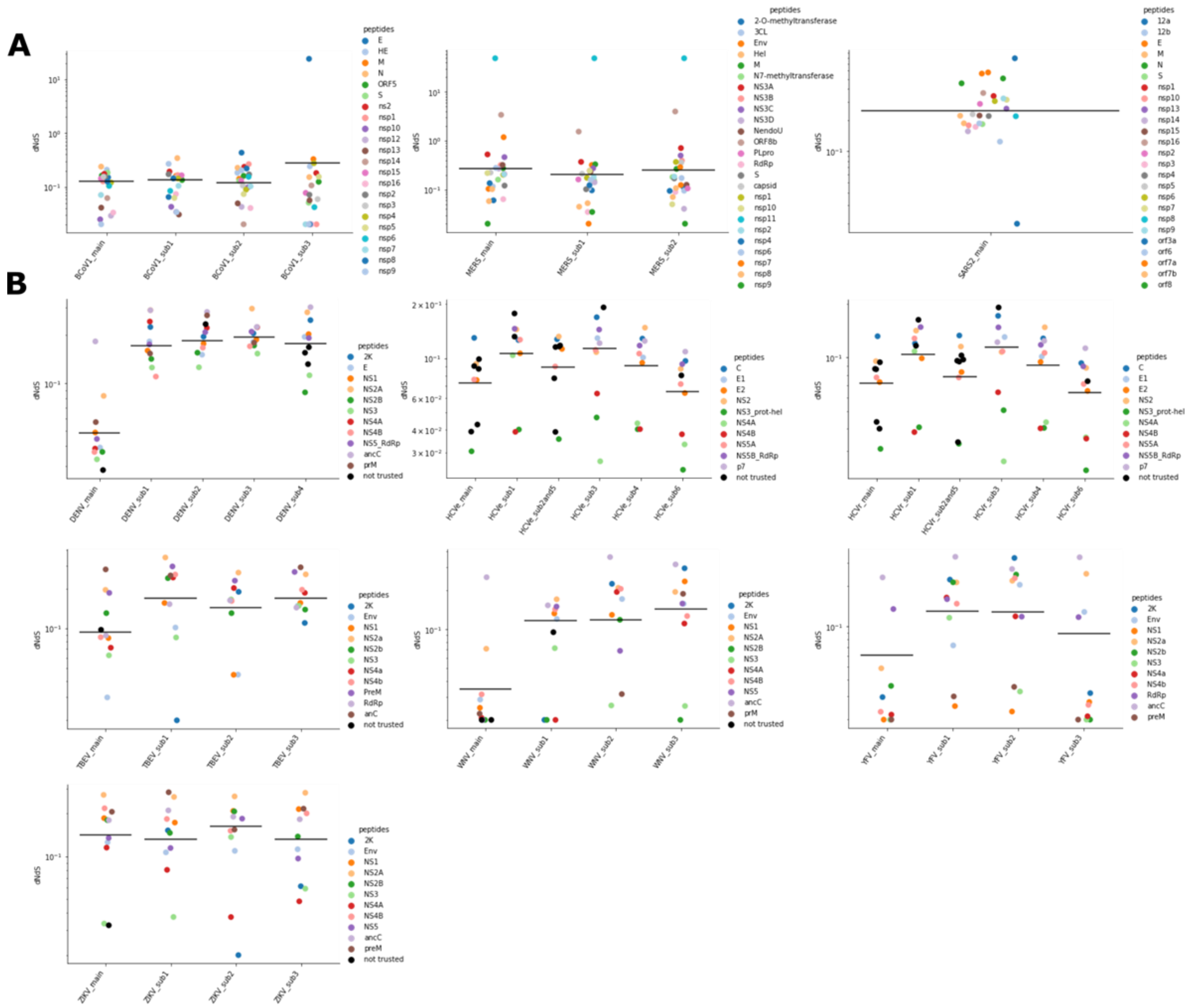

Fig. S15. *dN/dS* ratios retrieved from trees covering the whole population (corresponding to Figure S9) and trees representing manually selected lineages (correlating to viral sero- or genotypes). Each protein is represented by a single dot, black line represents mean over all proteins. Black coloured dots represent proteins with non-reliable *dN/dS* due to saturation of synonymous substitutions.

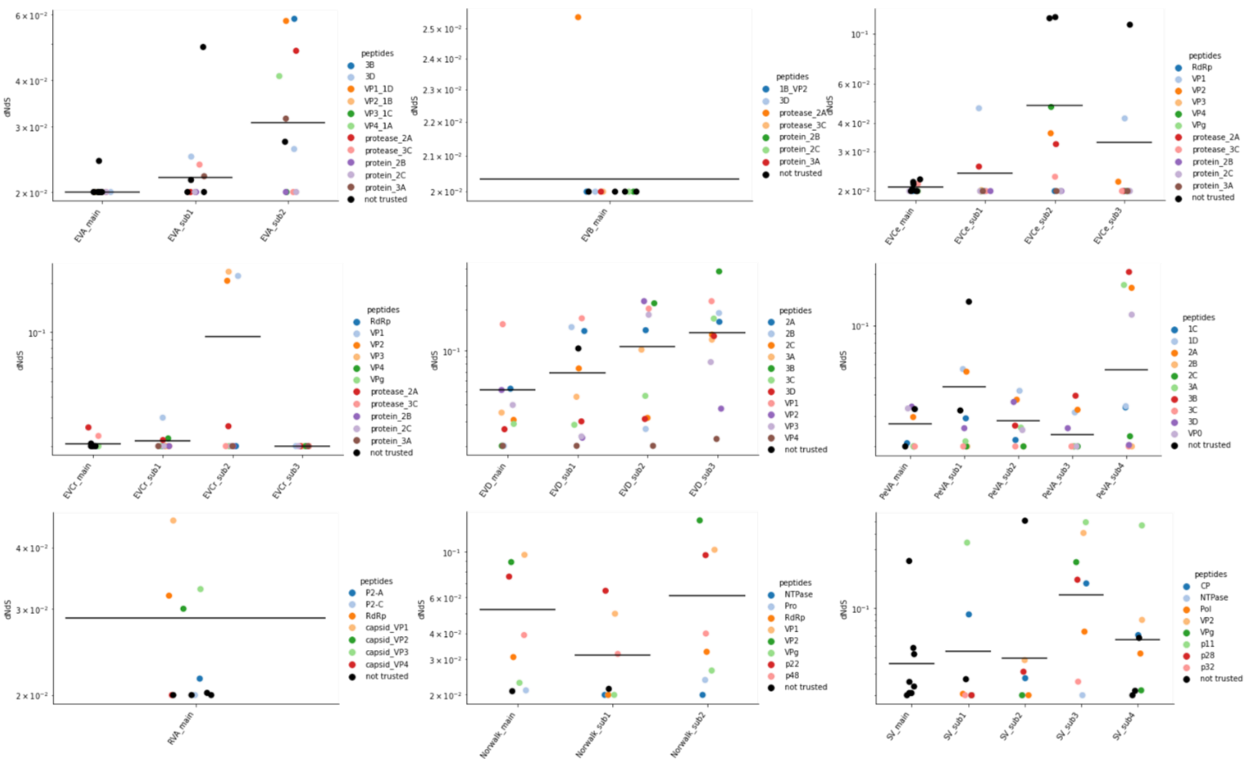

Fig. S16. *dN/dS* ratios retrieved from trees covering the whole population (corresponding to Figure S9) and trees representing manually selected lineages (correlating to viral sero- or genotypes). Each protein is represented by a single dot, black line represents mean over all proteins. Black coloured dots represent proteins with non-reliable *dN/dS* due to saturation of synonymous substitutions.

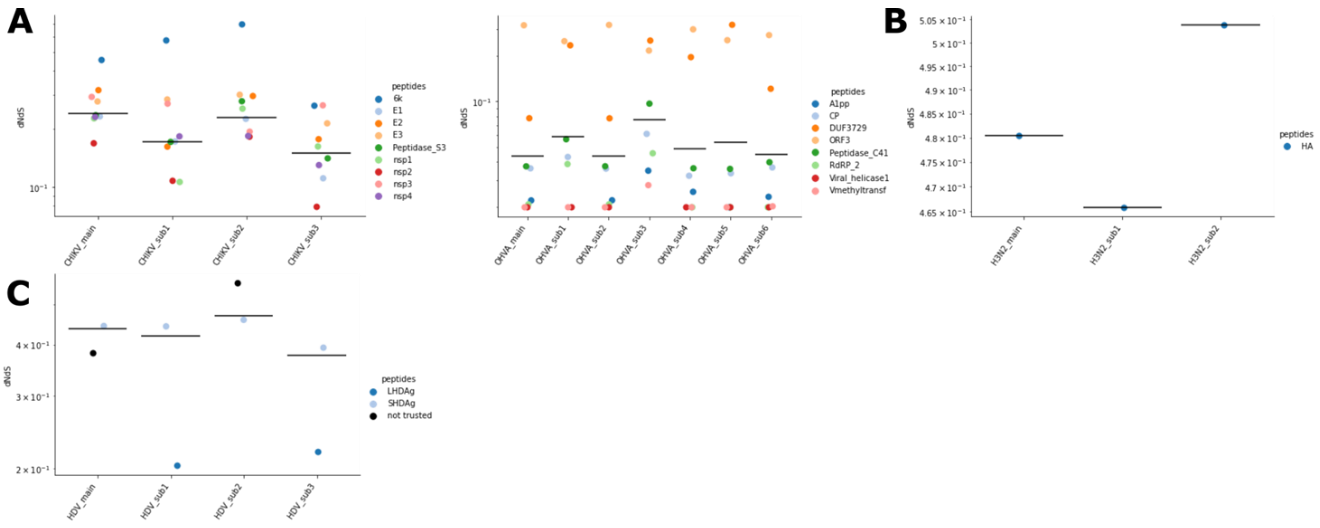

Fig. S17. *dN/dS* ratios retrieved from trees covering the whole population (corresponding to Figure S9) and trees representing manually selected lineages (correlating to viral sero- or genotypes). Each protein is represented by a single dot, black line represents mean over all proteins. Black coloured dots represent proteins with non-reliable *dN/dS* due to saturation of synonymous substitutions.

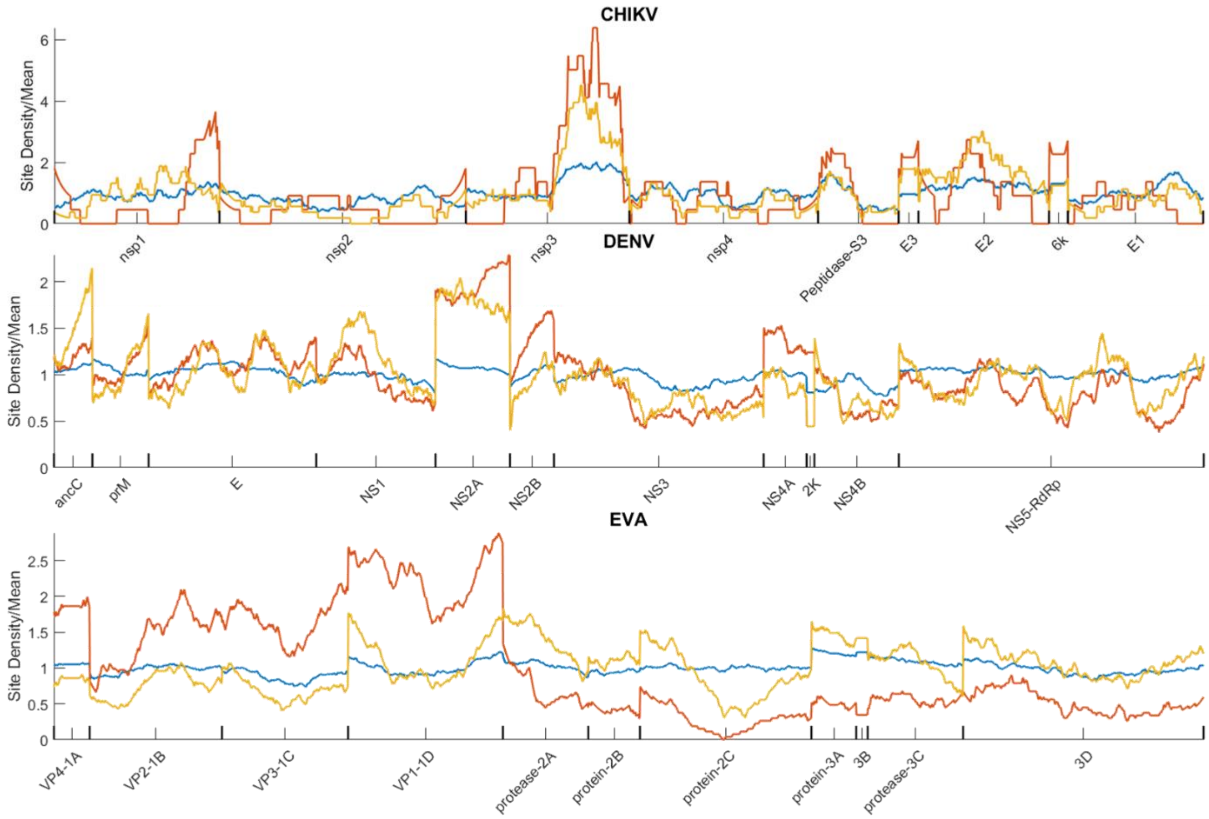

Fig. S18. The site density of three classes of mutations – Multiple, deep (MD, red), multiple, shallow (MS, yellow), and all shallow (AS, blue, see Methods and Figure 2) – computed over a sliding window of 101 amino acid sites respecting mature peptide boundaries and normalized with respect to the mean site density for each class over the entire genome.

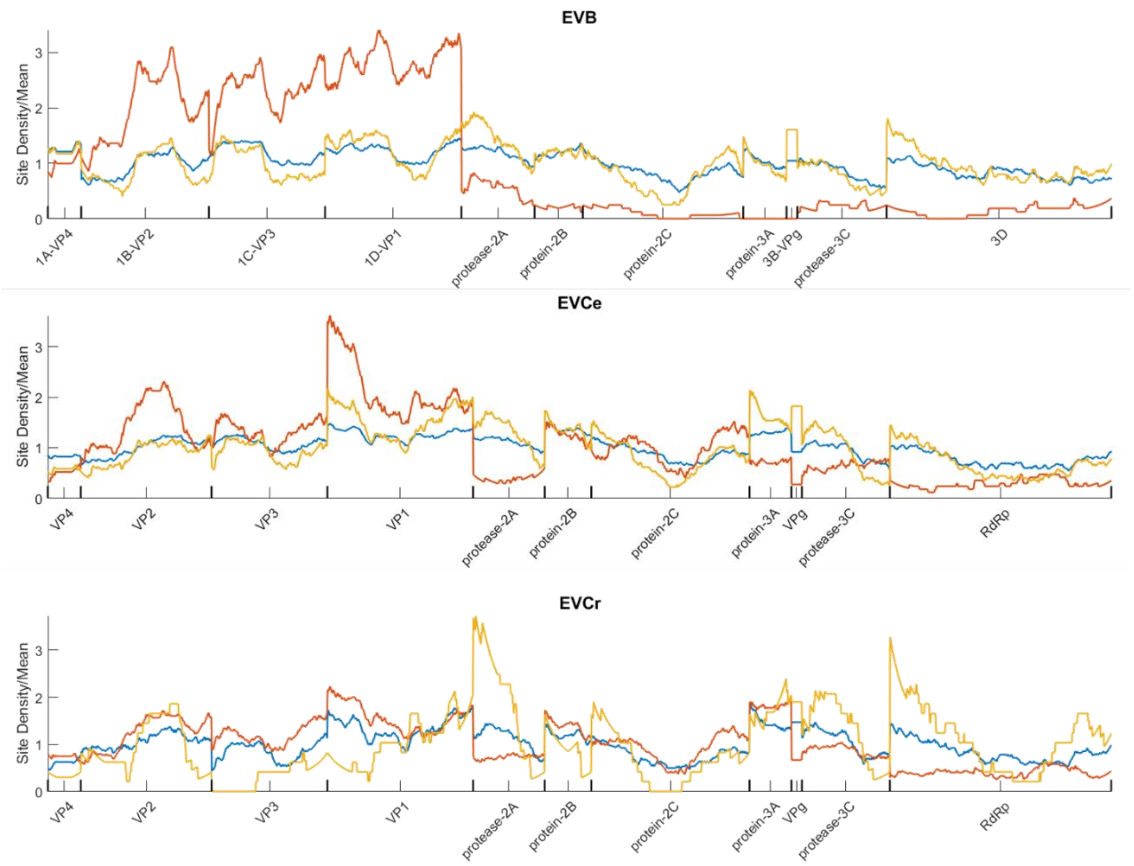

Fig. S19. The site density of three classes of mutations – Multiple, deep (MD, red), multiple, shallow (MS, yellow), and all shallow (AS, blue, see Methods and Figure 2) – computed over a sliding window of 101 amino acid sites respecting mature peptide boundaries and normalized with respect to the mean site density for each class over the entire genome.

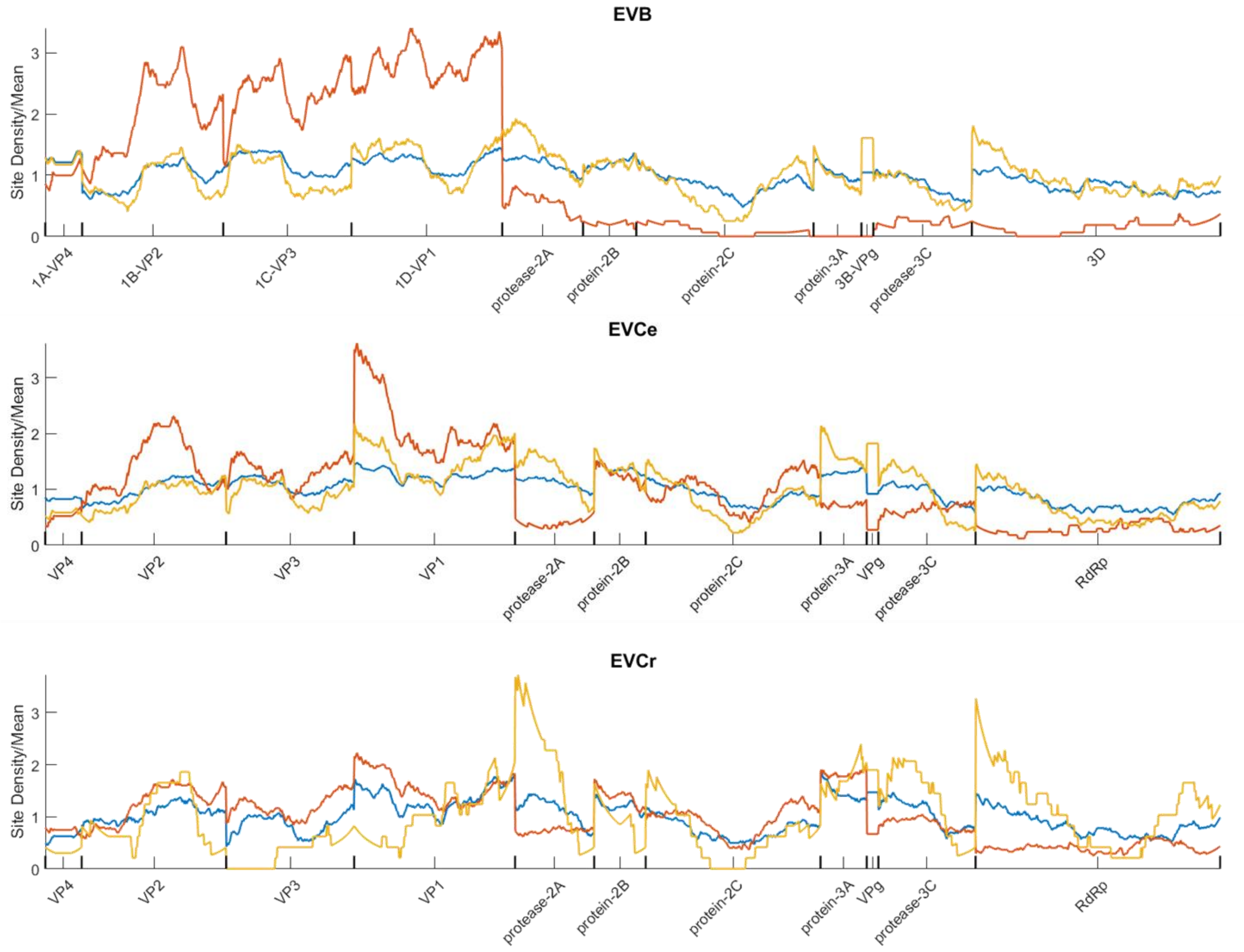

Fig. S20. The site density of three classes of mutations – Multiple, deep (MD, red), multiple, shallow (MS, yellow), and all shallow (AS, blue, see Methods and Figure 2) – computed over a sliding window of 101 amino acid sites respecting mature peptide boundaries and normalized with respect to the mean site density for each class over the entire genome.

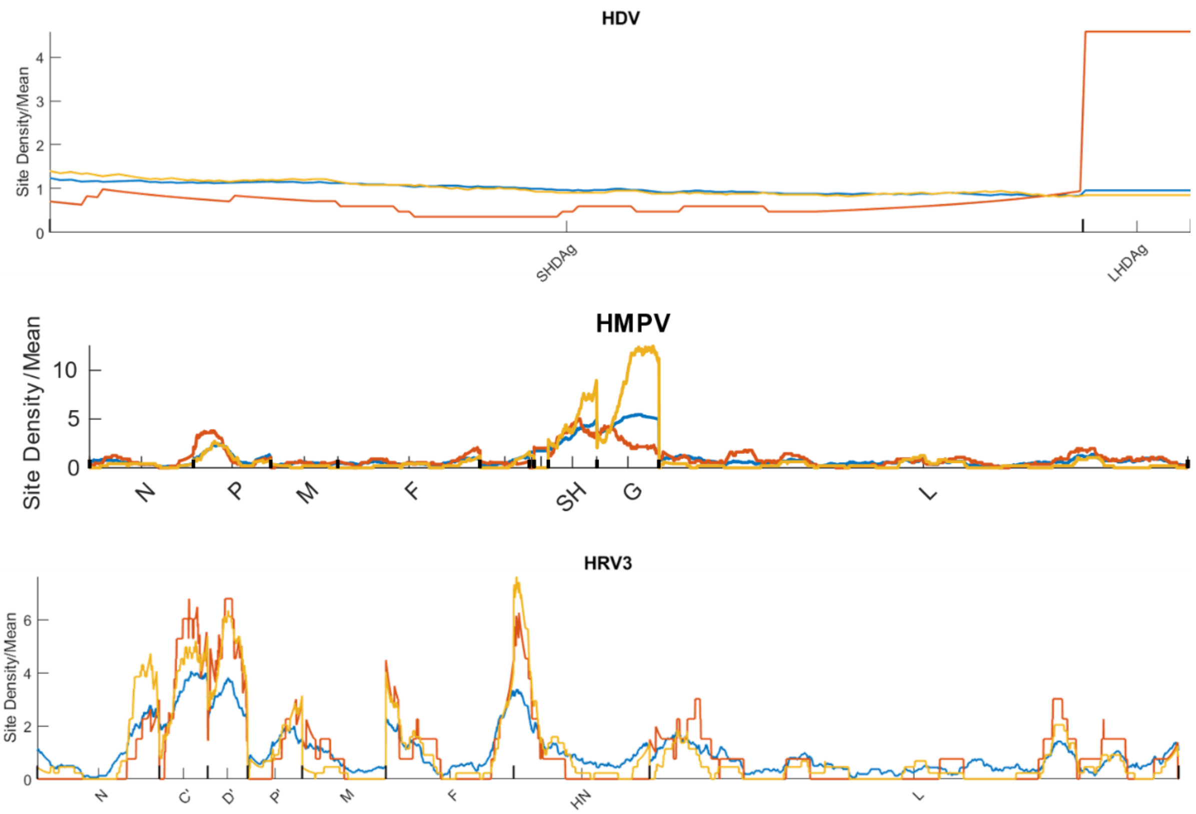

Fig. S21. The site density of three classes of mutations – Multiple, deep (MD, red), multiple, shallow (MS, yellow), and all shallow (AS, blue, see Methods and Figure 2) – computed over a sliding window of 101 amino acid sites respecting mature peptide boundaries and normalized with respect to the mean site density for each class over the entire genome.

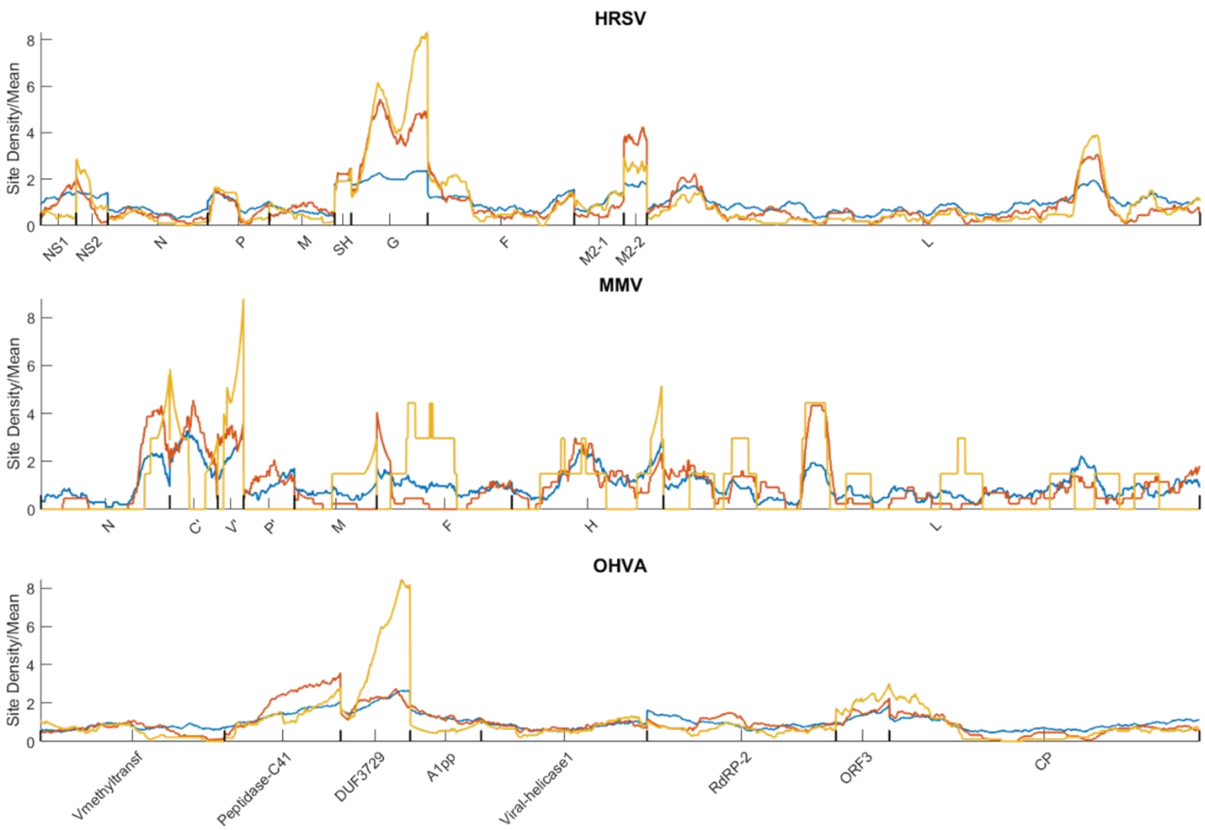

Fig. S22. The site density of three classes of mutations – Multiple, deep (MD, red), multiple, shallow (MS, yellow), and all shallow (AS, blue, see Methods and Figure 2) – computed over a sliding window of 101 amino acid sites respecting mature peptide boundaries and normalized with respect to the mean site density for each class over the entire genome.

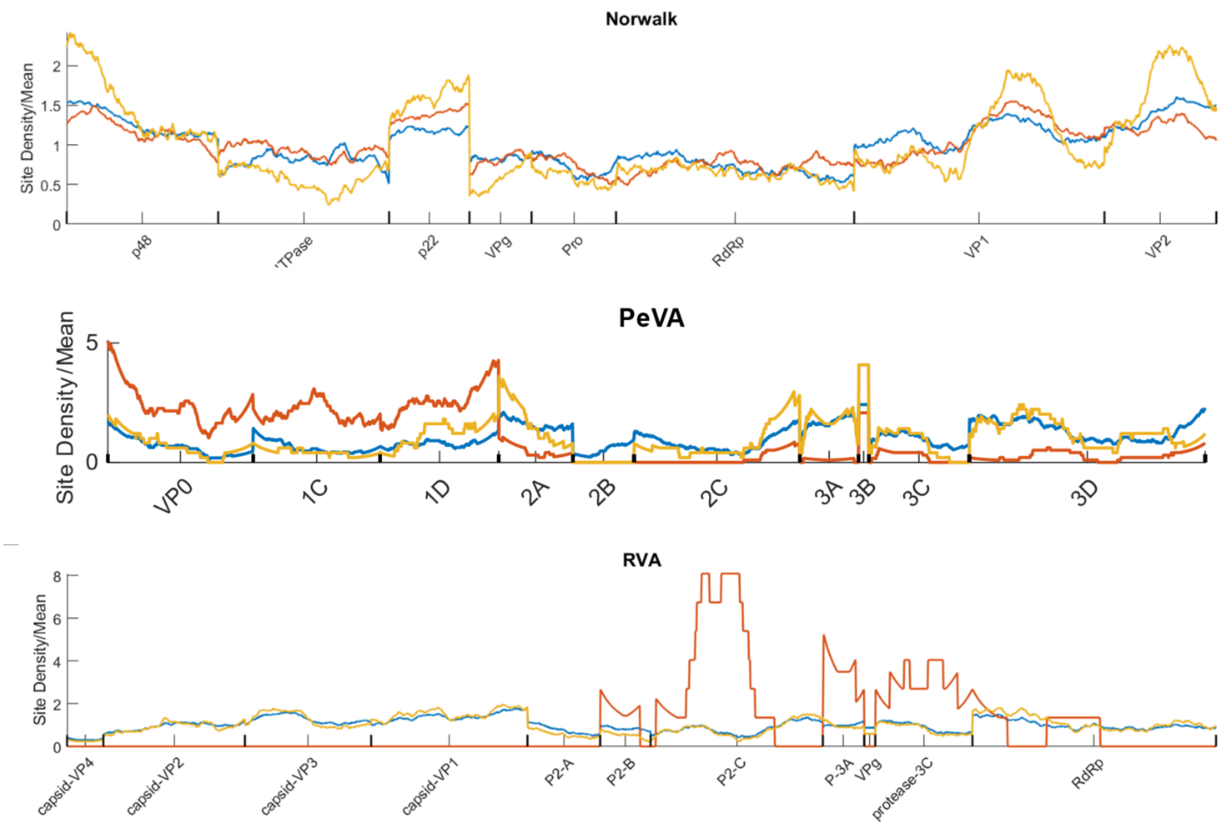

Fig. S23. The site density of three classes of mutations – Multiple, deep (MD, red), multiple, shallow (MS, yellow), and all shallow (AS, blue, see Methods and Figure 2) – computed over a sliding window of 101 amino acid sites respecting mature peptide boundaries and normalized with respect to the mean site density for each class over the entire genome.

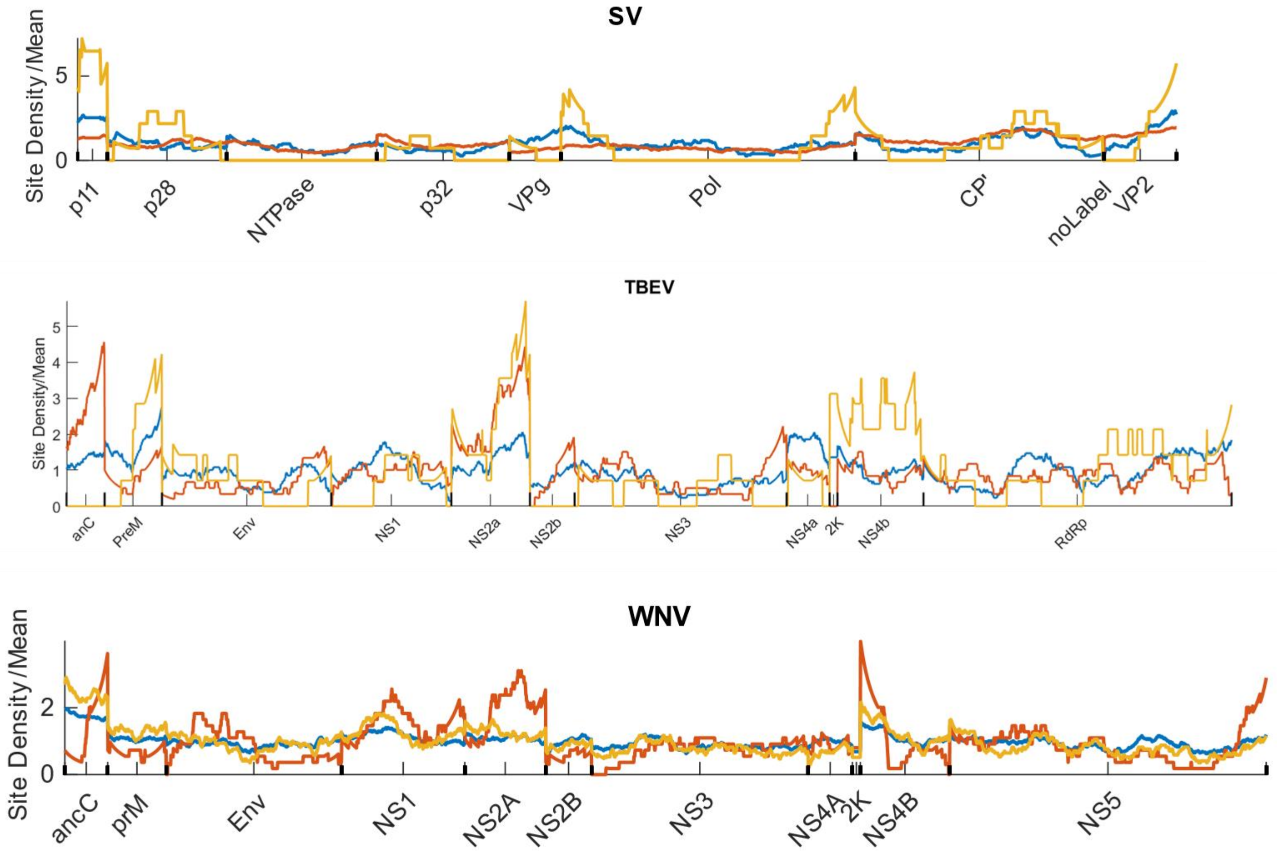

Fig. S24. The site density of three classes of mutations – Multiple, deep (MD, red), multiple, shallow (MS, yellow), and all shallow (AS, blue, see Methods and Figure 2) – computed over a sliding window of 101 amino acid sites respecting mature peptide boundaries and normalized with respect to the mean site density for each class over the entire genome.

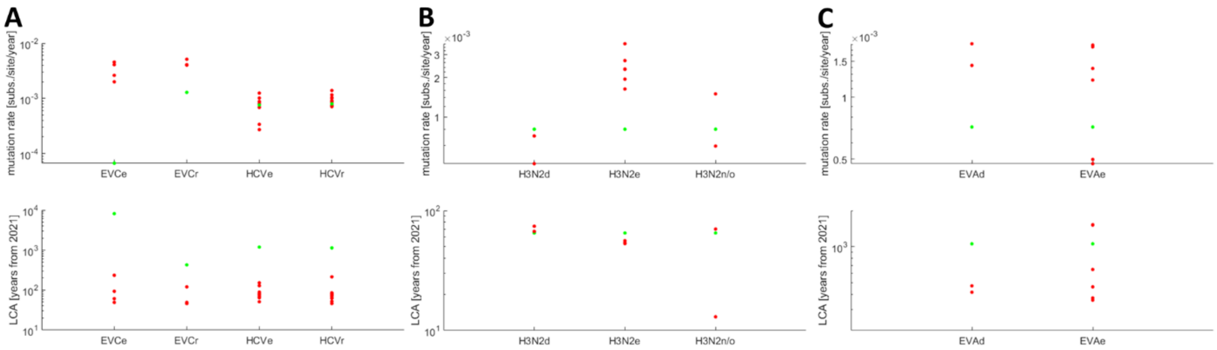

Fig. S25. Mutation rates (as substitutions per site per year) and estimated date of the last common ancestor for **A.** both HCV and EVC trees considered (e/r with and without manual pruning, see Methods). **B.** H3N2 diverse and evenly sampled subtrees (d/e) as well as new/old subtrees (n/o). **C.** EVA diverse and evenly sampled subtrees (d/e). Whole trees are shown green, subtrees in red.

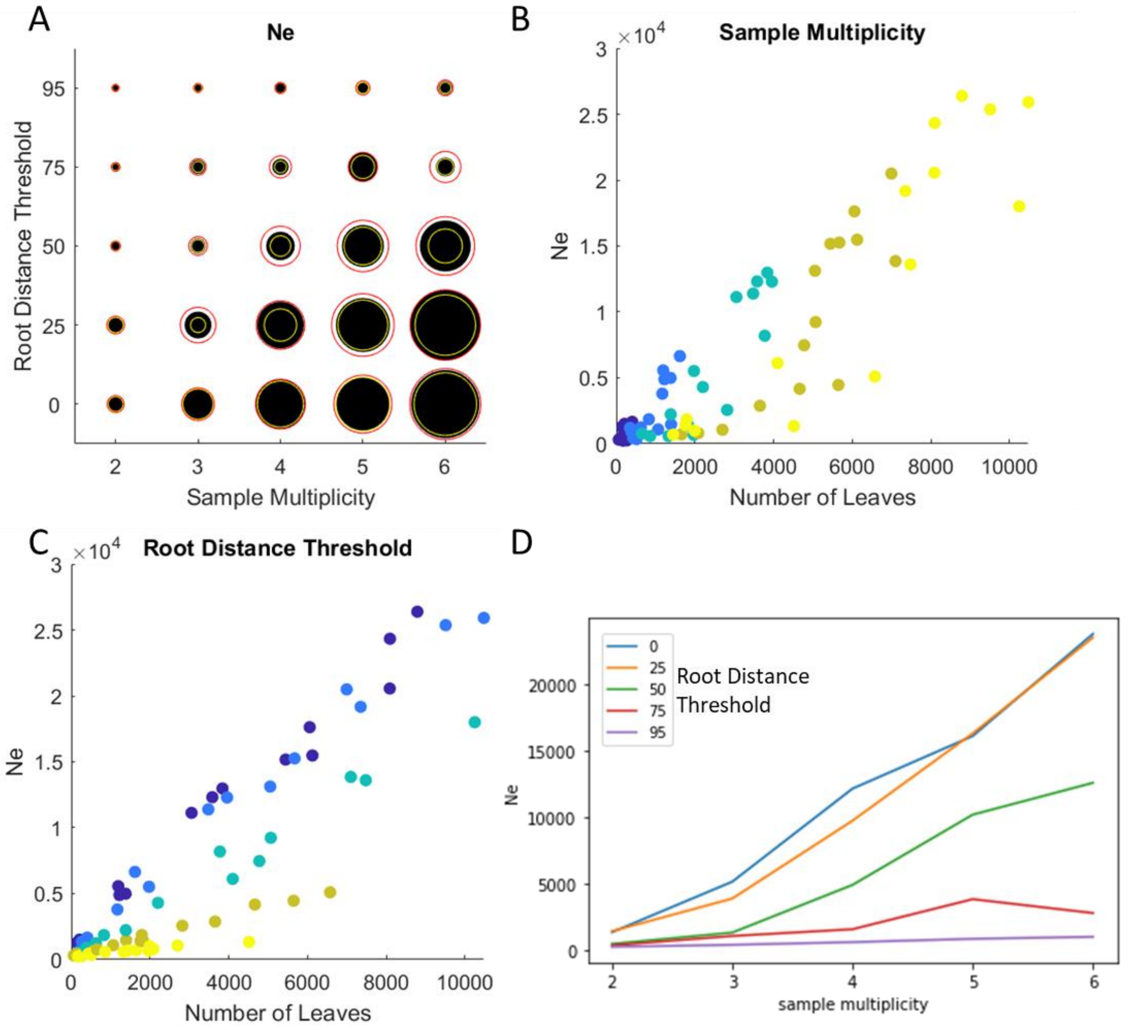

Fig. S26. A. Ne replicates for simulated trees, depending on sampling multiplicity and root distance threshold (representing selection). Largest repetition in red, middle in black and smallest in yellow. **B.** Sample multiplicity for each replicate of simulated trees plotted against *Ne* and number of leaves in the final tree. Sampling multiplicities shown in dark blue, light blue, green, brown, and yellow for multiplicities of 2, 3, 4, 5, and 6 respectively. **C.** Root distance threshold plotted against *Ne* and number of leaves of final simulated trees. Root distance thresholds shown in dark blue, light blue, green, brown, and yellow for thresholds of 0, 25, 50, 75 and 95, respectively. **D.** Increase of Ne with increased sampling density relative to selection strength (root distance thresholds 0, 25, 50, 75 and 95).

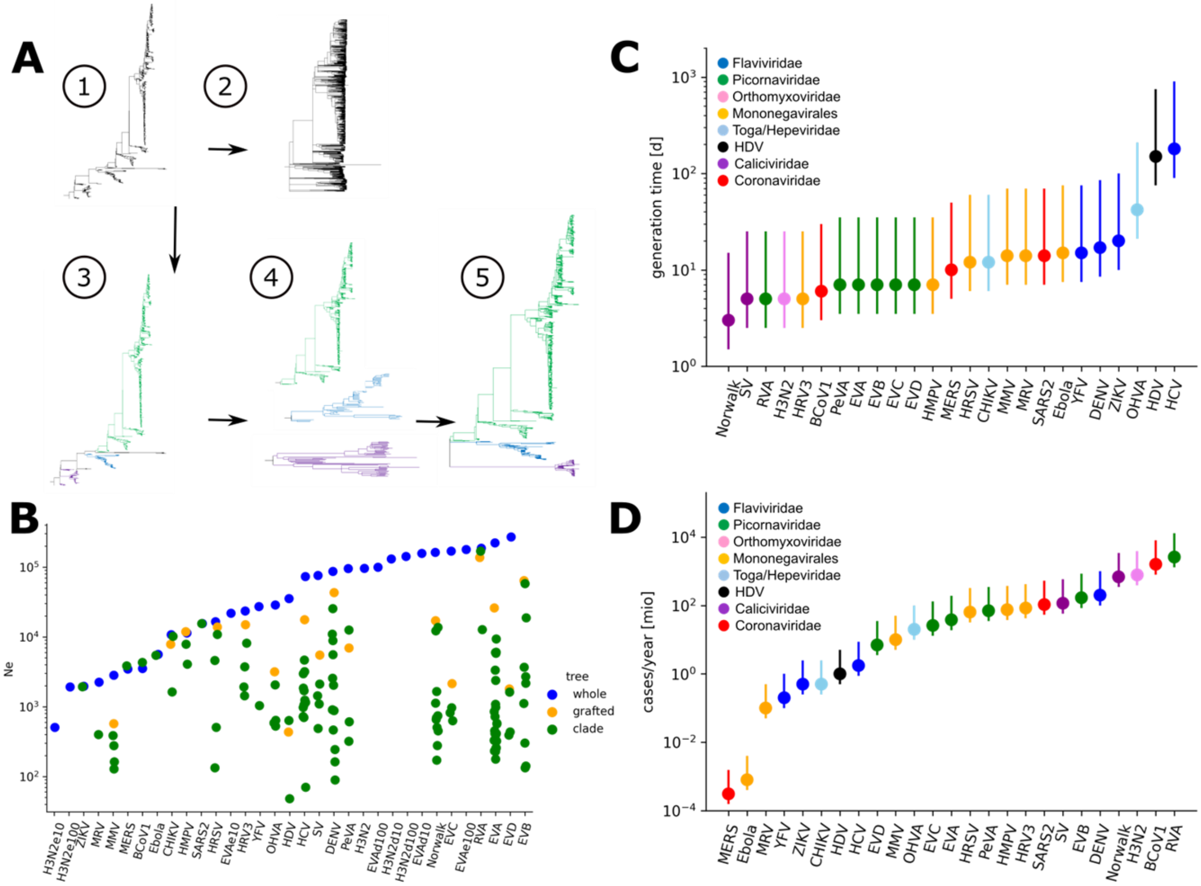

Fig. S27. A. Schematic of genealogical tree construction. Phylogenetic tree (1) which is either directly converted into genealogical tree (2) or split into clades (3) (as described in Fig. 2). Genealogical trees for each clade are calculated (4) and clades grafted at the root of oldest clade, keeping date of each leave intact (5). **B.** *Ne* estimates (according to Fisher-Wright model) for whole trees, clades and grafted trees. **C.** Estimated generation time *t* in days. Bars represent 0.5-5 times the value of the filled circle. **D.** Estimation of yearly viral cases. Bars represent 0.5-5 times the value of the filled circle.

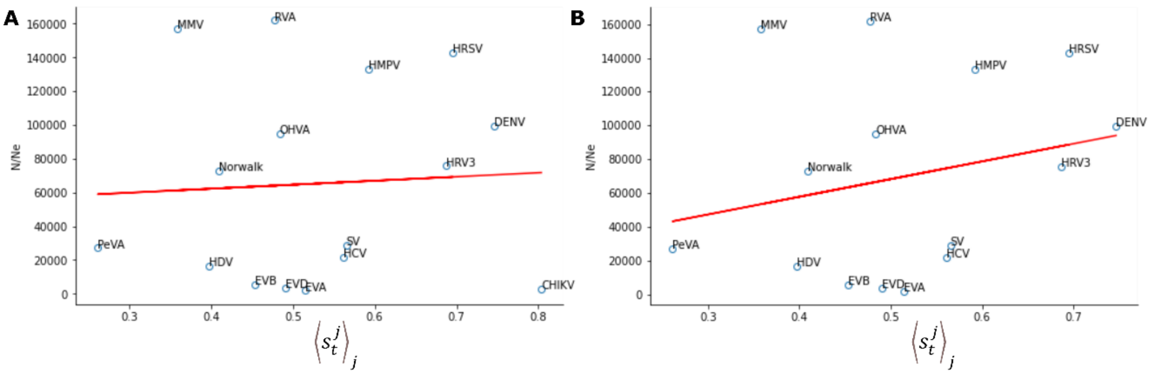

Fig. S28. S_t vs *N/Ne* with linear regression overlaid for all virus species showing more than 1 GL and where N/Ne could be estimated. **A.** Dataset including CHIKV. Regression curve: $y = 23641.3198x + 52823.2646$. Pearson's correlation coefficient r = 0.0587, p-value = 0.8291, standard error = 107520.8595. **B.** Dataset excluding CHIKV. Regression curve: $y = 104736.0124x + 15831.2877$. Pearson's correlation coefficient r = 0.2357, p-value = 0.3978, standard error = 119794.6123.

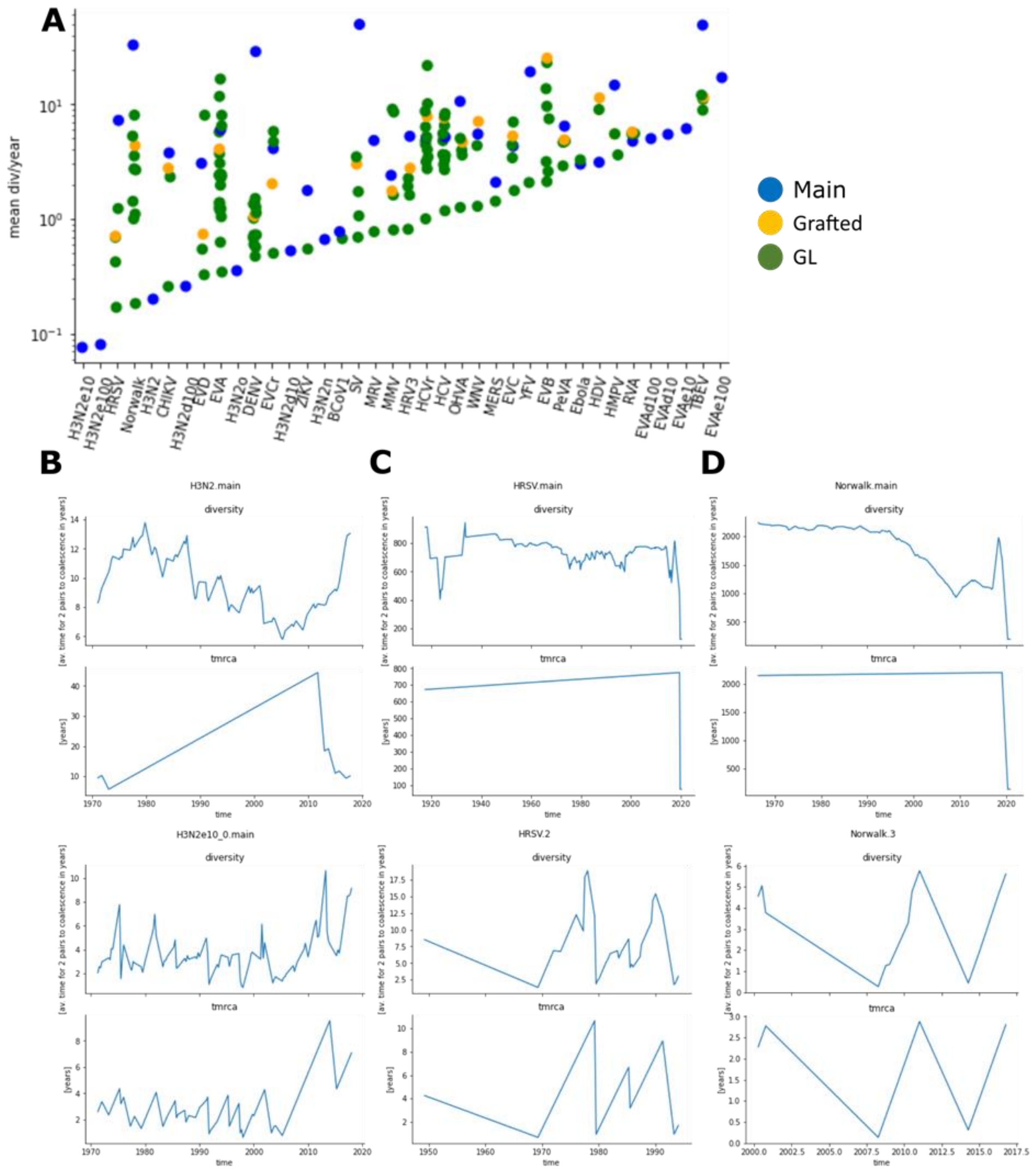

Fig. S29. A. Mean diversity per year based on genealogical trees for the main population, genealogical lineages (GL) and grafted trees. Diversity is calculated with PACT (the average time in years for any 2 pairs at a given timepoint to coalesce). **B-D**. Representative skyline plots for diversity and time to most recent common ancestor (TMRCA) of selected viral main populations or genealogical lineages. Plots for all sets can be found in the Supplementary data.

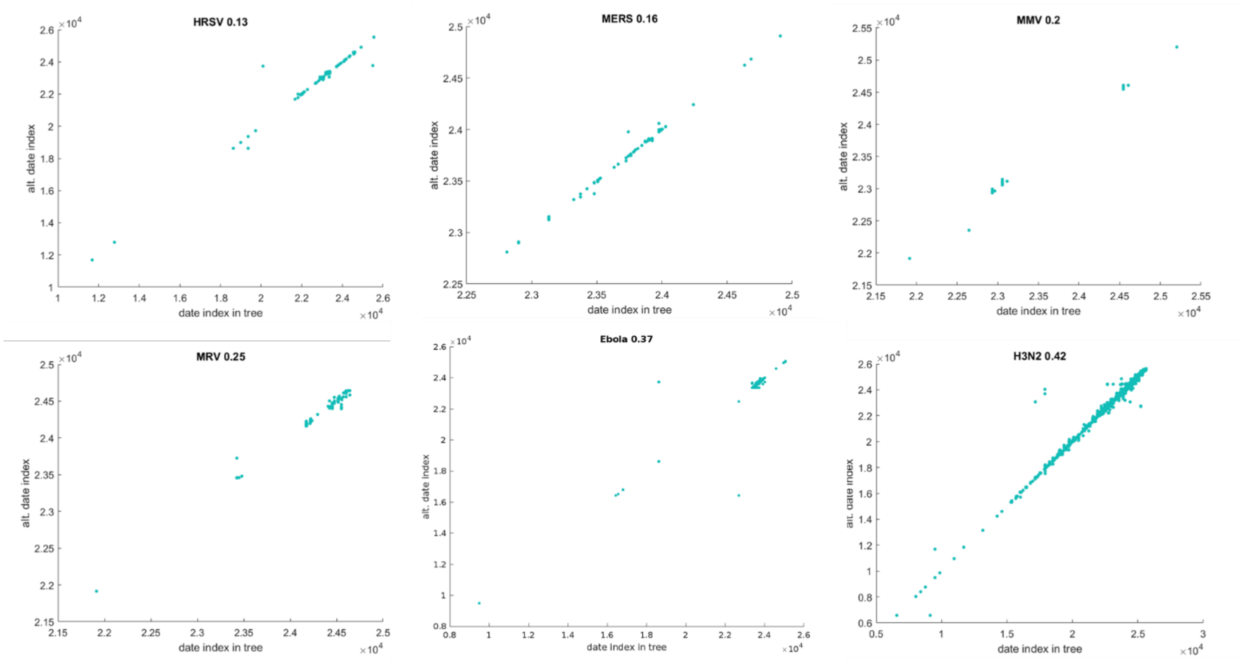

Fig. S30. The date of isolation for redundant isolates vs. the date of isolation for the leaf in the tree to which these sequences are mapped for all virus species where more than 10% of sequences are redundant. Isolates and leaves with no known date of isolation are excluded. Number next to virus species gives fraction of samples with redundancy.

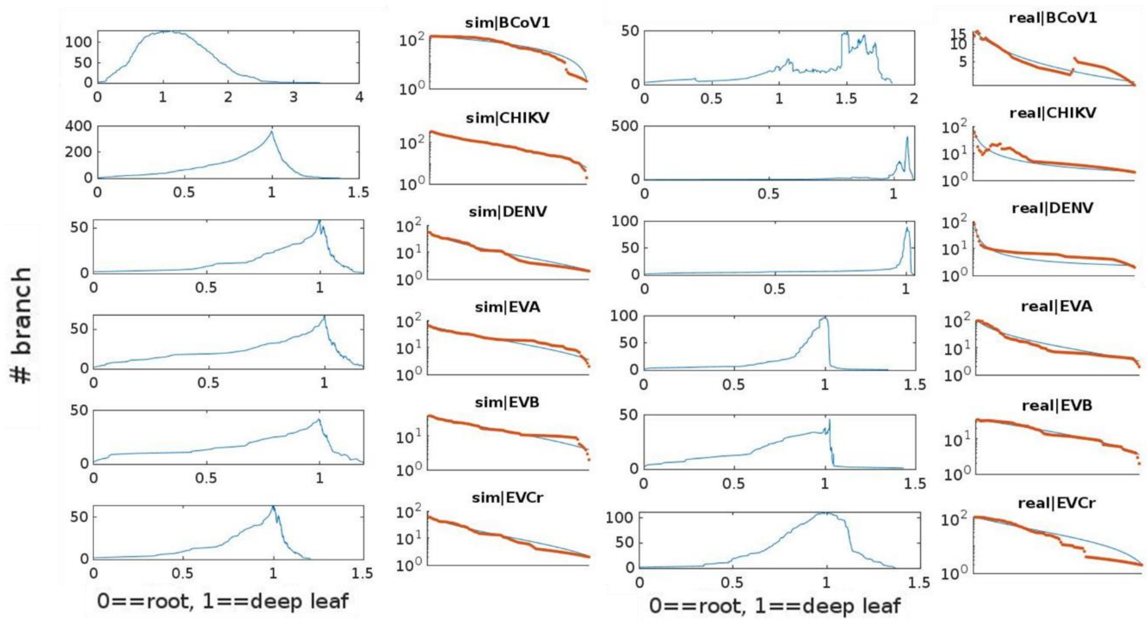

Fig. S31. *Left* The number of parallel branches of each (sub-sampled where appropriate) genealogical tree from the root [0] to the tree tips (deepest leaves [1]). *Right* The number of parallel branches moving from the deepest leaves back to the root overlaid with fitted power-law expression.

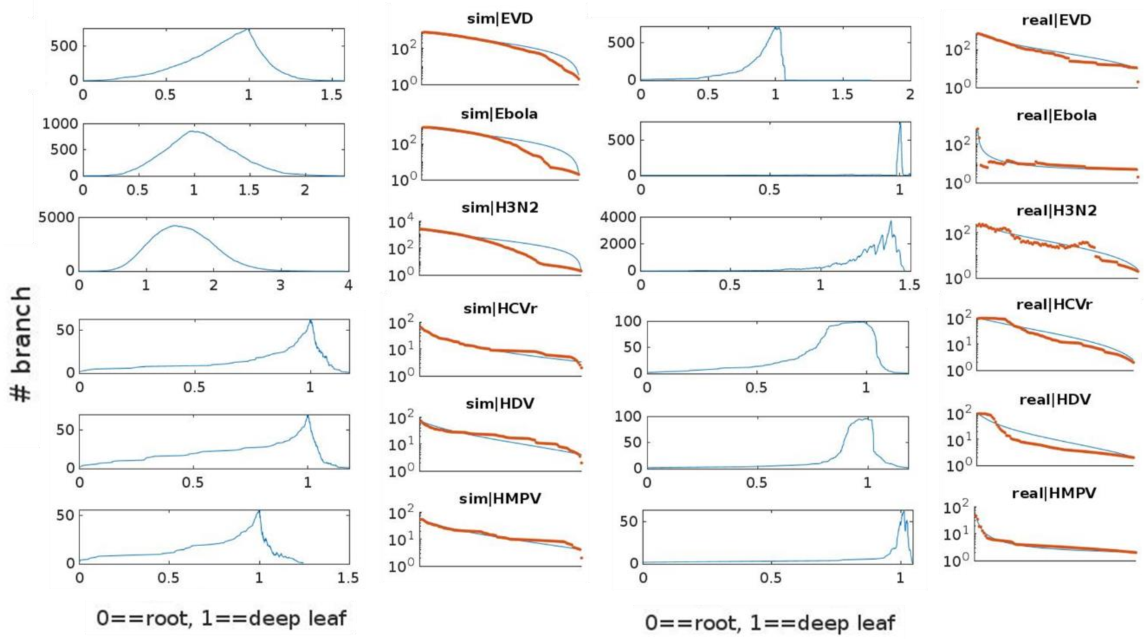

Fig. S32. *Left* The number of parallel branches of each (sub-sampled where appropriate) genealogical tree from the root [0] to the tree tips (deepest leaves [1]). *Right* The number of parallel branches moving from the deepest leaves back to the root overlaid with fitted power-law expression.

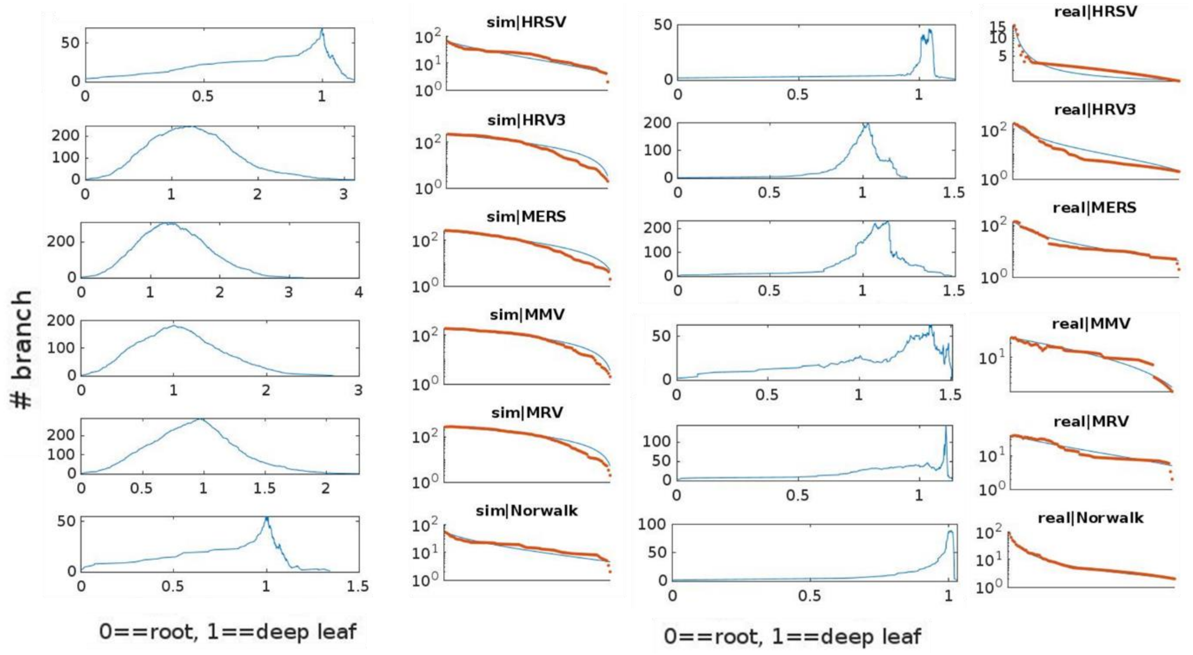

Fig. S33. *Left* The number of parallel branches of each (sub-sampled where appropriate) genealogical tree from the root [0] to the tree tips (deepest leaves [1]). *Right* The number of parallel branches moving from the deepest leaves back to the root overlaid with fitted power-law expression.

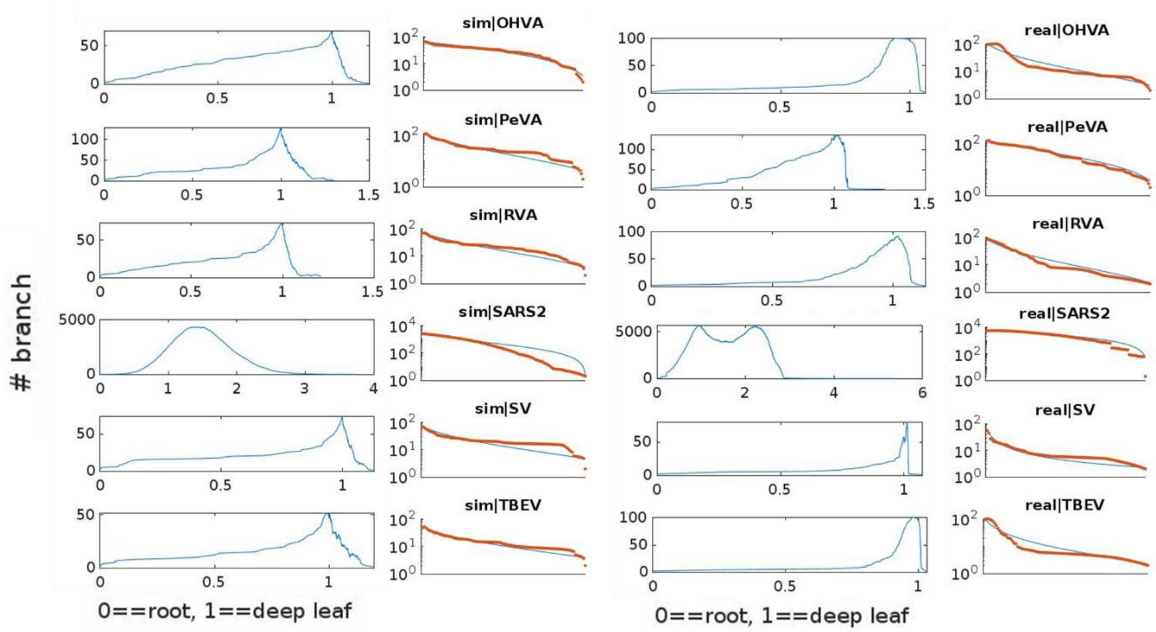

Fig. S34. *Left* The number of parallel branches of each (sub-sampled where appropriate) genealogical tree from the root [0] to the tree tips (deepest leaves [1]). *Right* The number of parallel branches moving from the deepest leaves back to the root overlaid with fitted power-law expression.

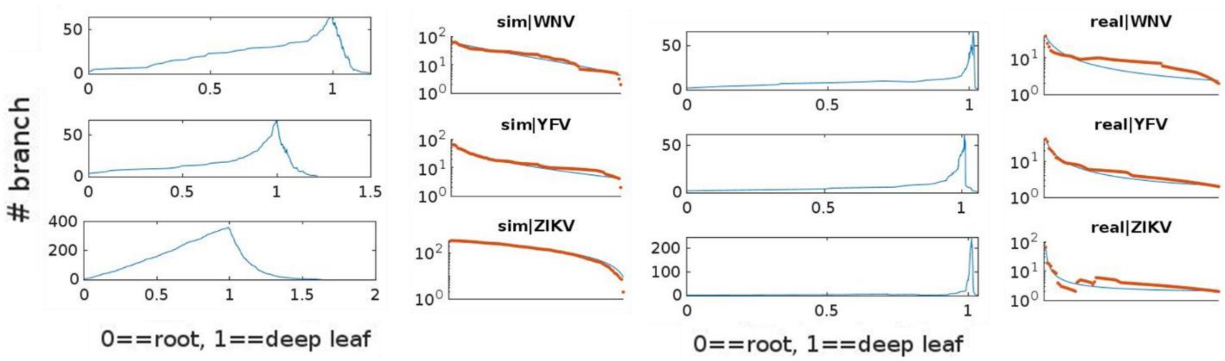

Fig. S35. *Left* The number of parallel branches of each (sub-sampled where appropriate) genealogical tree from the root [0] to the tree tips (deepest leaves [1]). *Right* The number of parallel branches moving from the deepest leaves back to the root overlaid with fitted power-law expression.

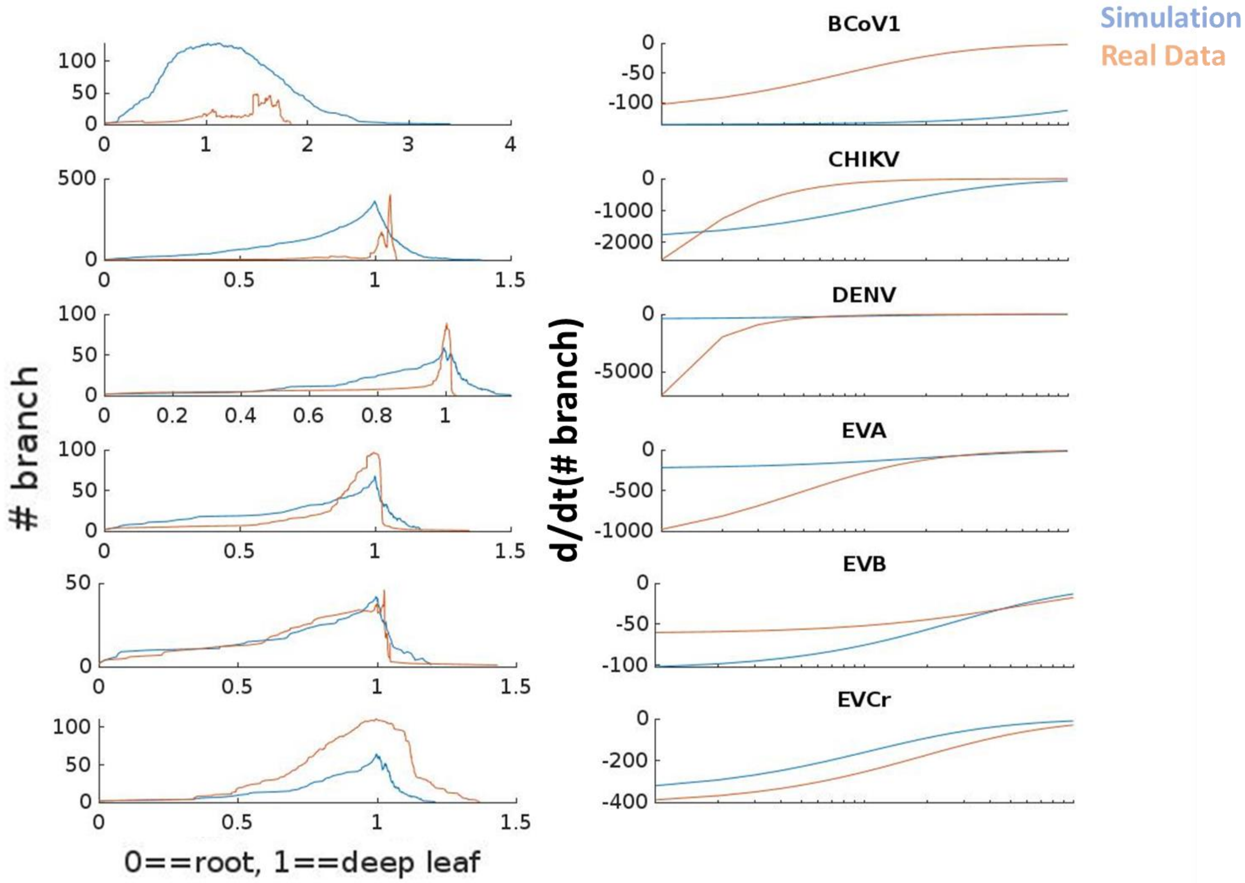

Fig. S36. *Left* The number of parallel branches of each (sub-sampled where appropriate) genealogical tree from the root [0] to the tree tips (deepest leaves [1]), real and simulated data overlaid. *Right* The time derivatives (of fitted power-laws) for the number of parallel branches moving from the deepest leaves back to the root.

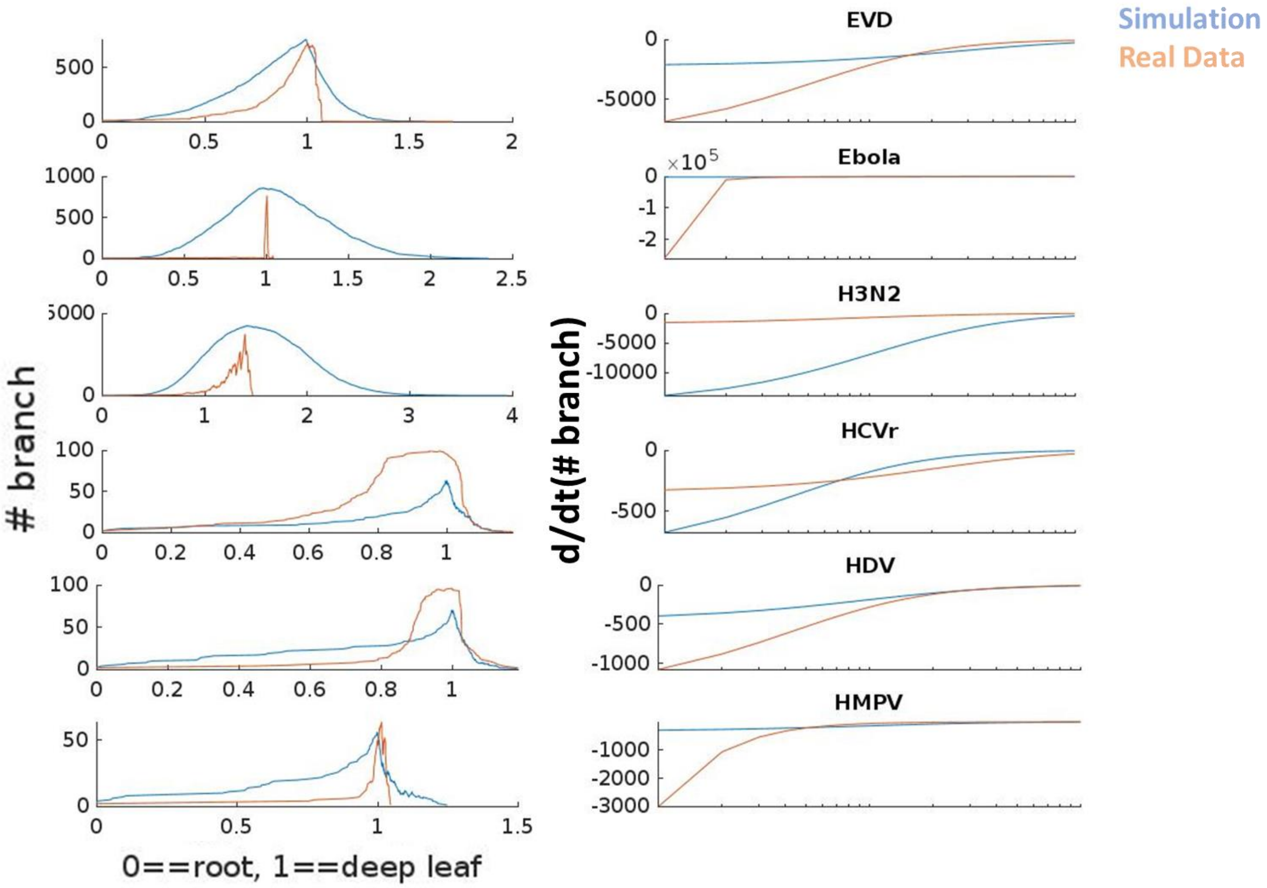

Fig. S37. *Left* The number of parallel branches of each (sub-sampled where appropriate) genealogical tree from the root [0] to the tree tips (deepest leaves [1]), real and simulated data overlaid. *Right* The time derivatives (of fitted power-laws) for the number of parallel branches moving from the deepest leaves back to the root.

.

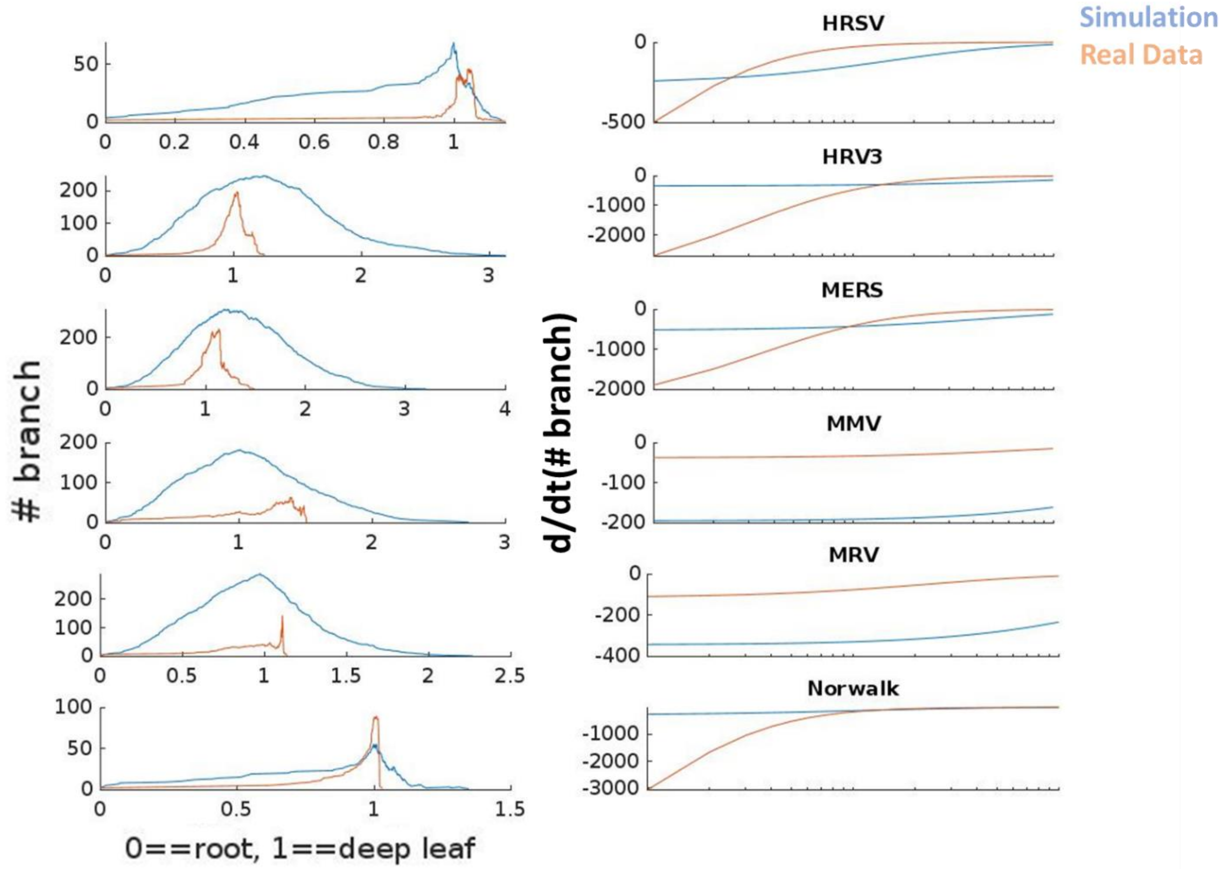

Fig. S38. *Left* The number of parallel branches of each (sub-sampled where appropriate) genealogical tree from the root [0] to the tree tips (deepest leaves [1]), real and simulated data overlaid. *Right* The time derivatives (of fitted power-laws) for the number of parallel branches moving from the deepest leaves back to the root.

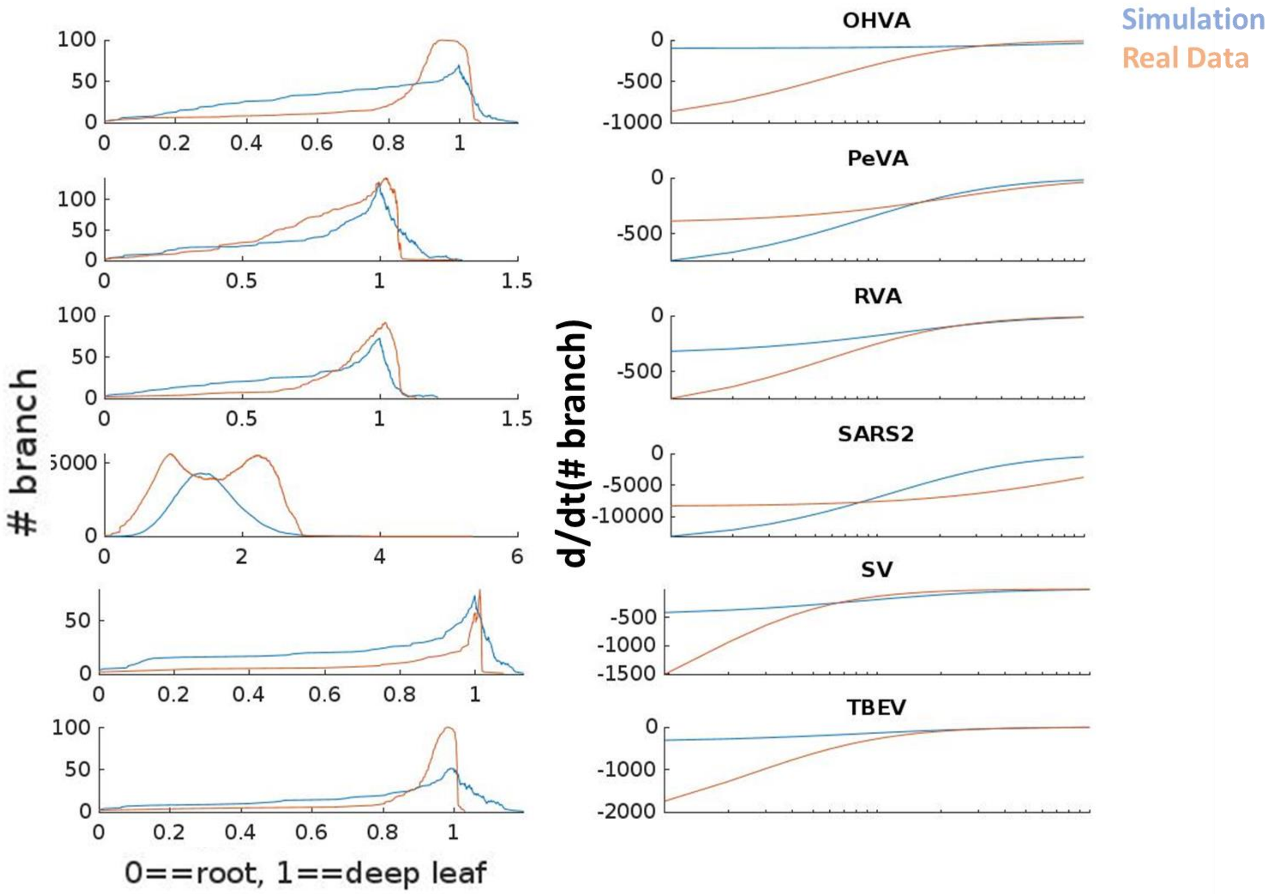

Fig. S39. *Left* The number of parallel branches of each (sub-sampled where appropriate) genealogical tree from the root [0] to the tree tips (deepest leaves [1]), real and simulated data overlaid. *Right* The time derivatives (of fitted power-laws) for the number of parallel branches moving from the deepest leaves back to the root.

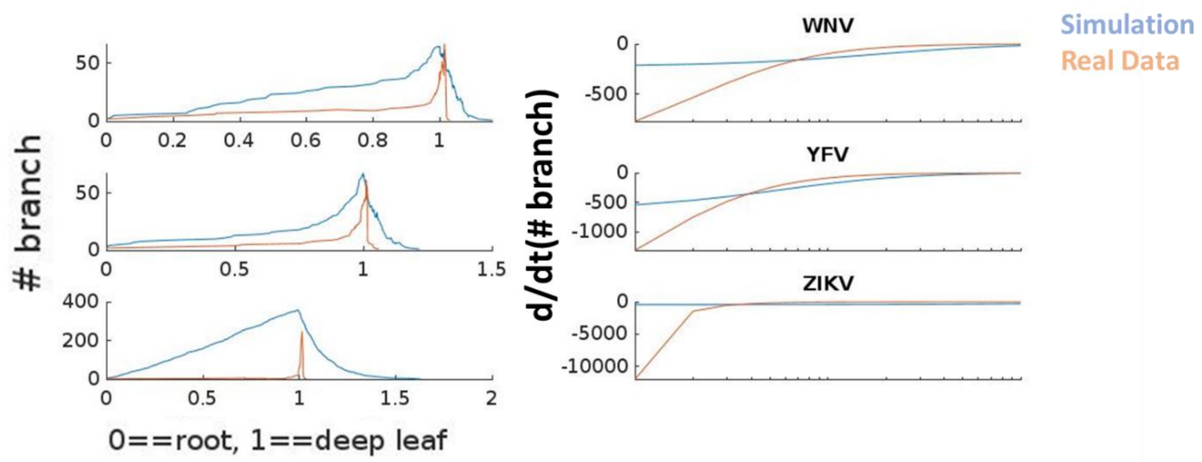

Fig. S40. *Left* The number of parallel branches of each (sub-sampled where appropriate) genealogical tree from the root [0] to the tree tips (deepest leaves [1]), real and simulated data overlaid. *Right* The time derivatives (of fitted power-laws) for the number of parallel branches moving from the deepest leaves back to the root.

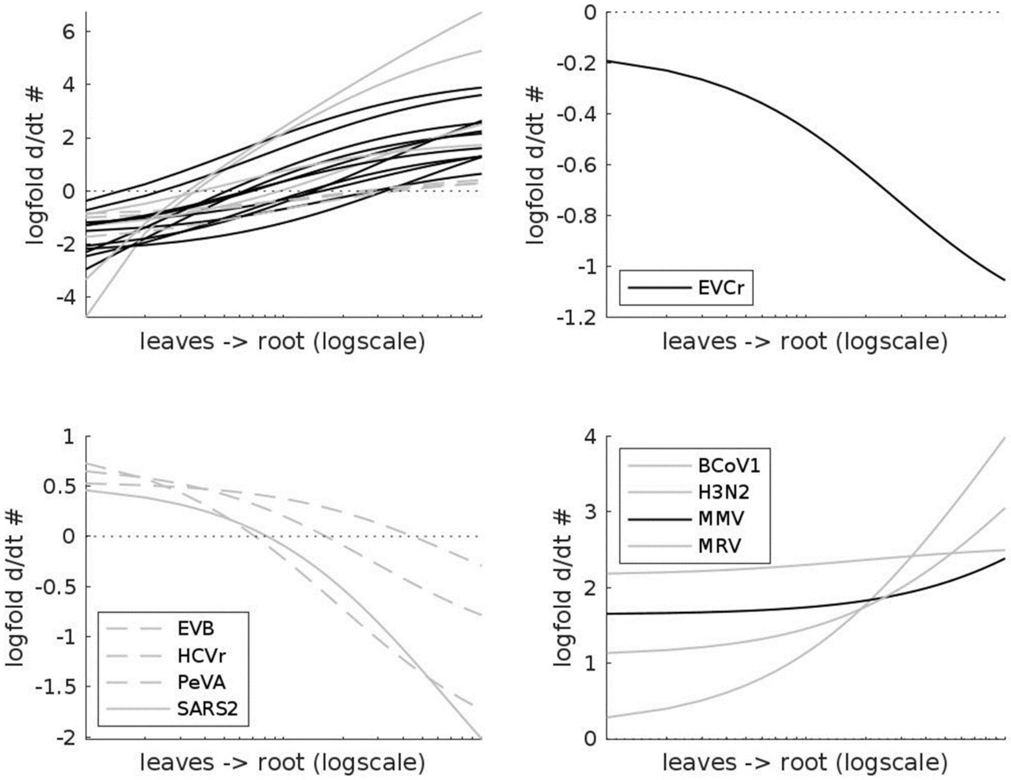

Fig. S41. Log-fold change (simulated/real) of the time derivatives for the number of parallel branches evaluated from the deepest leaves back towards the root grouped into 4 categories based on the sign at the deepest leaves and the root. Viruses containing 2 or more GLs with a correlation coefficient of 0.8 or above are displayed in solid, black; those without multiple GLs in sold, gray; and those with multiple GLs with a correlation coefficient below 0.8 in dashed, gray. Ebola, MERS, YFV, and ZIKA are displayed in the top left panel (solid gray) as well as HDV, RVA, and TBEV (dashed gray).

SI References

1. E. L. Hatcher *et al.*, Virus Variation Resource - improved response to emergent viral outbreaks. *Nucleic Acids Res* **45**, D482-d490 (2017).
2. Y. Bao *et al.*, The influenza virus resource at the National Center for Biotechnology Information. *J Virol* **82**, 596-601 (2008).
3. Y. Shu, J. McCauley, GISAID: Global initiative on sharing all influenza data–from vision to reality. *Eurosurveillance* **22**, 30494 (2017).
4. N. D. Rochman *et al.*, Ongoing global and regional adaptive evolution of SARS-CoV-2. *Proc Natl Acad Sci U S A* **118** (2021).
5. K. Katoh, K. Misawa, K. i. Kuma, T. Miyata, MAFFT: a novel method for rapid multiple sequence alignment based on fast Fourier transform. *Nucleic acids research* **30**, 3059-3066 (2002).
6. W. Li, A. Godzik, Cd-hit: a fast program for clustering and comparing large sets of protein or nucleotide sequences. *Bioinformatics* **22**, 1658-1659 (2006).
7. simplemaps (2021) World Cities Database.
8. L.-T. Nguyen, H. A. Schmidt, A. Von Haeseler, B. Q. Minh, IQ-TREE: a fast and effective stochastic algorithm for estimating maximum-likelihood phylogenies. *Molecular biology and evolution* **32**, 268-274 (2015).
9. M. N. Price, P. S. Dehal, A. P. Arkin, FastTree 2–approximately maximum-likelihood trees for large alignments. *PloS one* **5**, e9490 (2010).
10. T.-H. To, M. Jung, S. Lycett, O. Gascuel, Fast dating using least-squares criteria and algorithms. *Systematic biology* **65**, 82-97 (2016).
11. W. M. Fitch, Toward defining the course of evolution: minimum change for a specific tree topology. *Systematic Biology* **20**, 406-416 (1971).
12. Z. Yang, PAML 4: phylogenetic analysis by maximum likelihood. *Molecular biology and evolution* **24**, 1586-1591 (2007).
13. T. Bedford, S. Cobey, M. Pascual, Strength and tempo of selection revealed in viral gene genealogies. *BMC Evol Biol* **11**, 220 (2011).
14. Y. Ishida, A. Rosales, The origins of the stochastic theory of population genetics: The Wright-Fisher model. *Stud Hist Philos Biol Biomed Sci* **79**, 101226 (2020).
15. S. Vinh le, A. Fuehrer, A. von Haeseler, Random Tree-Puzzle leads to the Yule-Harding distribution. *Mol Biol Evol* **28**, 873-877 (2011).
16. G. U. Yule, II.—A mathematical theory of evolution, based on the conclusions of Dr. JC Willis, FR S. *Philosophical transactions of the Royal Society of London. Series B, containing papers of a biological character* **213**, 21-87 (1925).
17. C. Xue, Z. Liu, N. Goldenfeld, Scale-invariant topology and bursty branching of evolutionary trees emerge from niche construction. *Proc Natl Acad Sci U S A* **117**, 7879-7887 (2020).
